# Anodized Ti6Al4V-ELI, electroplated with copper is bactericidal against *Staphylococcus aureus* and enhances macrophage phagocytosis

**DOI:** 10.1007/s10856-024-06853-4

**Published:** 2025-01-24

**Authors:** Paula Milena Giraldo-Osorno, Adam Benedict Turner, Sebastião Mollet Barros, Robin Büscher, Simone Guttau, Farah Asa’ad, Margarita Trobos, Anders Palmquist

**Affiliations:** 1 Department of Biomaterials, Institute of Clinical Sciences, Sahlgrenska Academy, University of Gothenburg, Gothenburg, Sweden; 2 Centre for Antibiotic Resistance Research in Gothenburg (CARe), University of Gothenburg, Gothenburg, Sweden; 3 Stryker Trauma Gmbh, Schönkirchen, Germany; 4 Faculty of Medicine, Centre for Translational Bone, Joint and Soft Tissue Research, Technische Universität Dresden, Dresden, Germany; 5 Department of Oral Biochemistry, Institute of Odontology, Sahlgrenska Academy, University of Gothenburg, Gothenburg, Sweden

## Abstract

Implants aim to restore skeletal dysfunction associated with ageing and trauma, yet infection and ineffective immune responses can lead to failure. This project characterized the microbiological and host cell responses to titanium alloy with or without electroplated metallic copper. Bacterial viability counting and scanning electron microscopy quantified and visualized the direct and indirect bactericidal effects of the Cu-electroplated titanium (Cu-Ep-Ti) against two different *Staphylococcus aureus* strains. Human THP-1 macrophage adhesion and viability was analyzed, along with phagocytosis. Results showed potent antimicrobial activity alongside promising host-immunomodulatory properties. Direct and indirect exposure to Cu-Ep-Ti produced potent bactericidal effects resulting in 94–100% reductions in bacterial viability at 24 h, with complete eradication in some cases. As expected, cytotoxicity was observed in THP-1 macrophages without media exchange, though when media was exchanged at 8, 24 and 48 h cell viability was equivalent to Control-Ti. Interestingly macrophages adhered to the copper material or grown in the presence of copper ions showed 7-fold increase in phagocytosis of *S. aureus* bioparticles compared to Control-Ti, suggesting a dual bactericidal and host immunomodulatory mechanism. In conclusion, this Cu-electroplated Ti biomaterial can limit bacterial contamination on the implant surface, whilst simultaneously promoting a beneficial antimicrobial immune response.

**Graphical Abstract:**

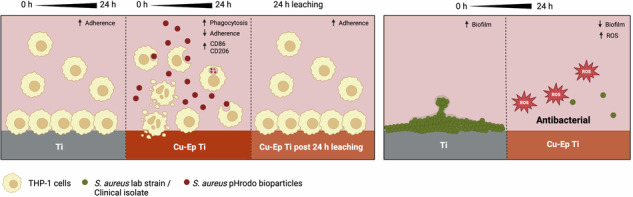

## Introduction

Bone-anchored implanted medical devices are a common surgical tool used to restore or prevent the loss of orthopedic function associated with ageing and/or physical trauma [1]. However, these devices are not resistant to infection and their biological properties can be improved to mitigate infection risk. Infection and host rejection leading to loss of orthopedic devices is an uncommon but debilitating consequence of orthopedic device implantation [2–5]. While implant loss is multifactorial, septic failure due to infection is among the most common etiologies [6–8]. The incidence of infection can vary greatly depending on the site and type of implantation, ranging from 2 to 4% for primary and revision joint arthroplasty to as high as 23% for fracture fixation pins [8–10]. A point of concern is that the frequency of joint arthroplasties continues to increase annually, and infection increases proportionally [11]. An increase in infection rate, in combination with the ongoing threat and forecasted development of antimicrobial resistance indicates an urgent need for improved technologies to prevent periprosthetic joint infection (PJI) as these significantly imperil the potential for successful implant and patient survival now and in the future [12–14]. However, as of yet, no material designed for long-term internal implantation is completely resistant to infection [15].

Many approaches have been investigated to produce materials resistant to infection, however, the implant microenvironment is incredibly complex – and the introduction of antimicrobial elements can cause significant biological dysfunction [16]. As such, when designing the next generation of orthopedic biomaterials, any antimicrobial inclusion must be biocompatible, or better, provide beneficial immunostimulatory properties that improve healing or osseointegration. The approach of balancing antimicrobial and host beneficial properties of the next generation of implanted biomaterials has been under investigation for some time, and various approaches are currently being researched [17]. These include topographical modifications, altering surface chemistry, application of surface coatings, and the alloying of bioactive elements, among others [16, 17]. One such approach is the electrodeposition of elements such as copper onto the surface of existing biomaterials.

Copper is an attractive metal inclusion as the biological effects induced are plethoric [18, 19]. Various antimicrobial mechanisms of action have been attributed to copper [18, 20], therefore, the incorporation of metallic copper onto a biomaterial surface to endow it with antimicrobial properties has the potential to prevent biomaterial-associated infection (BAI) [18]. In addition, copper is also a fundamental micronutrient for the proper functioning of the immune system, with copper deficiency being linked to infection susceptibility [21]. The application of exogenous copper has also been shown to exert great effects on the host immune response and could act synergistically with the known antimicrobial benefits to provide the material with immunostimulatory properties. For example, copper treatment has been found to modulate in vitro murine macrophage elimination of *Escherichia coli* [20, 21]. An effect that was inhibited by the application of antioxidants, suggesting that copper modulates the generation of bactericidal reactive oxygen species (ROS) by macrophages. Further, the application of copper chelators has been shown to increase the intracellular survival of bacteria within macrophages, implying that in addition to the modulation of ROS, copper plays an important role in the effective elimination of intracellular pathogens [22, 23]. Recent studies have also shown that the presence of copper plays a role in the coordination of various aspects of the immune response. Copper nanospheres were observed to induce an inflammatory response in macrophages, increasing the production of key pro-inflammatory cytokines including interleukin-6 (IL-6), interleukin-1β (IL-1β), interferon-γ (IFN-γ) and tumor necrosis factor-α (TNF-α) [20, 24].

Combined, the current knowledge of copper and its interactions with pathogen and host suggests that the addition of copper to orthopedic biomaterials may present intrinsic antimicrobial properties and immunostimulatory modulation of the immune system to eliminate microorganisms. This study aims to evaluate if copper when integrated onto Ti6Al4V-ELI limits bacterial viability and modulates the immune system contributing to bacterial eradication by enhancing phagocytosis. This in vitro study provides a proof-of-concept of the bactericidal and immunomodulatory functions of a novel copper-eluting biomaterial.

## Materials and methods

### Material fabrication, copper deposition, and characterization

Commercial Ti6Al4V-ELI disks (⌀ 12 mm, height 3 mm) were sourced from Stryker Trauma GmbH (Schönkirchen, Germany). The surface modification was developed and carried out by DOT GmbH (DOT GmbH, Rostock, Germany). All specimens were anodized in 20% sodium hydroxide. The control specimens were blasted with glass pearls (⌀ 40–70 µM). The test specimens were processed by electrochemical deposition of metallic copper and finally blasted with glass pearls. The final concentration of copper was measured to 0.6 ± 0.3 µg/mm^2^ by X-ray fluorescence (one measurement/sample). All samples were sterilized by gamma irradiation (min. 25 kGy) in accordance with ISO 11137. Within this work, unplated Ti6Al4V-ELI is referred to as Control-Ti and copper-electroplated Ti6Al4V-ELI is referred to as Cu-Ep-Ti.

#### Material surface characterization

Surface morphology and elemental composition were evaluated by scanning electron microscopy (SEM) and energy dispersive X-ray spectroscopy (SEM-EDX) using a Zeiss Leo Gemini 1530 (Zeiss, Oberkocken, Germany) operating at 20 kV. The surface wettability was assessed by the sessile water droplet contact angle method using a drop shape analyzer (DSA 100E, KRÜSS GmbH, Germany). Static contact angles of water droplets (4.2 μL) were measured on a balanced horizontal stage at room temperature. Images were recorded for 5 s after initial contact to allow for stabilization before measuring the final contact angle. Three water droplets were measured in distinct regions of each sample. The surface roughness was measured and mapped using a stylus profilometer (DektakXT; Bruker, Germany). The profile length measured was 1400 µM and the parameter measured was average roughness (Pa). The software used (Vision64) by DektakXT is in accordance with ISO 4287.

For contact angle investigation, four independent samples were tested. For roughness parameter investigation, five and nine independent samples were tested for the Control-Ti and Cu-Ep-Ti respectively.

#### Ion release

The release of Cu-ions was evaluated through sequential inoculation and measurement of Cu-Ep-Ti and Control-Ti samples in 1 mL Tryptic Soy Broth (TSB; Millipore, Sigma-Aldrich, USA) or Roswell Park Memorial Institute (RPMI) 1640 (Thermo Fisher Scientific, Waltham, USA). The samples were measured at 1, 10, 20, 40 min, 1, 2, 4, 8, 24, 48, 72, 96, 120, 144, and 168 h. At each time point, growth media was removed, and fresh media was added for subsequent measurements. To quantify ion release from the samples, the stored media were diluted (1:10) in HNO_3_ and analyzed using ICP-OES (PlasmaQuant 9000, Analytik-Jena, Endress+Hauser Company, Germany) with AspectPQ software. The ion release assays were carried out at 37 °C with 5% CO_2_. Three independent samples (*n* = 3) were tested with two technical replicates.

### Culture conditions

#### *Staphylococcus aureus* culture conditions

*Staphylococcus aureus* ATCC 25923 (American Type Culture Collection, Rockville, MD, USA) and an *S. aureus* clinical isolate from periprosthetic joint infection (PJI) (M19.373/MIC6942) [25] were streaked from −80 °C stocks onto 5% Horse Blood Columbia agar (HBA) plates (Media Department, Clinical Microbiology Laboratory, Sahlgrenska University Hospital, Gothenburg, Sweden) and incubated aerobically overnight at 37 °C. For inoculum preparation in all microbiological experiments, single colonies were added to 4 mL saline (0.9%) to achieve an OD_546_ of 0.13 (equivalent to 10^8^ CFU/mL) using a colorimeter (Sherwood Chroma 260, Cambridge, UK), and further diluted into tryptic soy broth (TSBg) (Scharlau, Barcelona, Spain) or RPMI 1640 supplemented with 10% heat-inactivated fetal bovine serum (HI-FBS) (Sigma-Aldrich, Saint Louis, USA).

#### THP-1 cell culture conditions

The human THP-1 monocytic cell line ATCC TIB-202 (Rockville, MD, USA) was grown in T75 cell culture treated flasks (Thermo Fisher Scientific, Waltham, USA) in RPMI supplemented with 10% HI-FBS, 0.5% β-mercaptoethanol (Sigma-Aldrich, Saint Louis USA) and 1% penicillin/streptomycin (PEST) solution (Gibco Life Technologies, Waltham, USA) in a 37 °C humidified incubator with 5% CO_2_. Cells between passages 5 and 9 were used and medium was changed every 2 days.

### Direct adhesion of *S. aureus* and THP-1 macrophages on material surfaces

“Direct” refers to the seeding and adhesion of either *S. aureus* or THP-1 macrophages on the surface of a material sample. All direct adhesion experiments were performed with 3 independent biological experiments (*n* = 3) and 2 technical replicates.

#### *S. aureus* biofilm seeding on material surfaces

To evaluate the bactericidal properties of the Cu-Ep-Ti, 1 mL inoculum of *S. aureus* ATCC 25923 or M19.373 in either TSBg or RPMI (10^5^ CFU/mL) was added to the Control-Ti or Cu-Ep-Ti surface in a 24-well microtiter plate (Thermo Fisher Scientific, Waltham, USA) and incubated statically at 37 °C for 4 h and 24 h. At each time point, the planktonic *S. aureus* cells were transferred to a sterile 24-well microtiter plate and re-incubated at 37 °C, under shaking (125 rpm) for 24 h to investigate the effect of prolonged copper exposure on the viability of *S. aureus* ATCC 25923 and M19.373.

##### Viable colony counting of *S. aureus*

Upon completion of the desired incubation periods, viable colony-forming unit (CFU) counting was performed differently depending on whether adhered biofilm or planktonic bacteria were counted. For adhered biofilm bacteria, material samples were gently rinsed three times in sterile saline (0.9%) and transferred to a 15 mL centrifuge tube (Sarstedt, Nümbrecht, Germany) containing 1 mL saline. The samples were then sonicated (42 kHz) (Branson 3510MT Ultrasonic Cleaner, Brookfield, USA) for 30 s and vortexed at 10,000 rpm for 1 min. The detached bacterial cells were serially diluted (10-fold) in saline supplemented with 0.1% Triton-X100 (Thermo Fisher Scientific, Waltham, USA) before plating 5 µL spots on blood agar plates for CFU enumeration. For planktonic bacteria, samples were resuspended by pipetting and serially diluted 10-fold in saline and 0.1% Triton-X100 before spot-plating on blood agar plates for CFU enumeration.

##### Scanning electron microscopy (SEM) of surface-adhered *S. aureus* biofilms

One mL (10^5^ CFU/mL) *S. aureus* ATCC 25923 or M19.373 in either TSBg or RPMI was added to each well of a 24-well plate containing either the Control-Ti or the Cu-Ep-Ti and incubated statically at 37 °C for 4 h or 24 h. Discs were then rinsed three times in saline, and *S. aureus* cells adhered to the surfaces were fixed with 4% formaldehyde (HistoLab AB, Askim, Sweden) for 1 h at room temperature (RT) followed by dehydration at 4 °C in a graded ethanol series (50%–70%–80%–90%–95%–100%) for 5 min at each concentration, with the final 100% step being repeated once. After dehydration, *S. aureus* cells adhered to the sample surfaces were gold sputtered (10 nm) using a Leica EM ACE600 Sputter Coater (Leica, Stockholm, Sweden) and imaged in secondary electron mode using a LEO 55 ULTRA FEG SEM (Zeiss, Oberkochen, Germany) operating at 5 kV.

#### THP-1 macrophage culturing on material surfaces

THP-1 cells were seeded directly on top of either material surface in a 24-well microtiter plate (Thermo Fisher Scientific, Waltham, USA) at a density of 500,000 cells in 2 mL RPMI and stimulated with 10 ng/mL phorbol-12-myristate-13-acetate (PMA) (Sigma-Aldrich, Burlington, USA) for 24 h to differentiate them into macrophages. After this, the quantification of Cu ions released from each sample was performed using ICP-OES as described in “*S. aureus* biofilm seeding on material surfaces”.

##### Cell viability of THP-1 cells on material surfaces

To evaluate cell viability 24 h after seeding, THP-1 cells were detached from the surfaces with trypsin-EDTA (0.25%; Gibco Life Technologies, Waltham, USA) and collected in a centrifuge tube. A NucleoCounter® NC-100TM (ChemoMetec A/S, Lillerød, Denmark) system was used to determine total cell count and non-viable cell count. Cell viability was carried out following the manufacturer’s guidelines. In short, for quantifying non-viable cells, detached THP-1 cells were loaded into a Nucleocassette™ pre-coated with fluorescent propidium iodide, which stains cell nuclei. To determine the total cell count, 50 µL of the detached cell suspension were treated with NucleoCounter® lysis (50 µL) and stabilization buffer (50 µL) and subsequently loaded into a Nucleocassette™. Viable cells were then quantified as:

$${Viable\; cell\; number}={Total\; cell\; number}-{Non\; viable\; cell\; number}$$

##### THP-1 cell phagocytosis of *S. aureus* bioparticles on material surfaces

Phagocytosis was quantified using pHrodo™ red *S. aureus* bioparticles (Thermo Fisher Scientific, Roskilde, Denmark), which allows for the visualization and quantification of phagocytosed pHrodo *S. aureus* bioparticles. The bioparticles are nonfluorescent at neutral pH outside of the cell, but internalization in the phagolysosomes decreases pH, thereby inducing fluorescence. Cell culture media was discarded, and macrophages adhered for 22 h on either Control-Ti or Cu-Ep-Ti were stimulated with 500 µL of pHrodo™ red *S. aureus* bioparticles diluted into Live Cell Imaging Solution (Invitrogen, Waltham, MA, USA). After 2 h of incubation at 37 °C, fluorescence was measured at 544/590 nm using a FLUOstar Omega Microplate reader (BMG LABTECH, Ortenberg, Germany). The fluorescence intensity (FI) from the pHrodo bioparticles was normalized to cell viability to account for any cytotoxicity caused by the materials.

To ensure that the observed fluorescence was due to phagocytosis and not media acidification caused by the presence of copper ions, corresponding acidification controls were included, as shown in Fig. S1 (in supplementary information). For these controls, 500 µL of pHrodo™ Red S. aureus bioparticles diluted in Live Cell Imaging Solution was added to: (1) THP-1 macrophages (Positive control), (2) 24 h conditioned media from pre-leached Cu-Ep-Ti (without cells), (3) 24 h conditioned media from pre-leached Control Ti (without cells), and (4) THP-1 macrophages grown 24 h in conditioned media from pre-leached Cu-Ep Ti, where one centrifugation step was added to remove cells. This setup ensured that any changes in fluorescence were specific to phagocytosis rather than the acidifying effect of the copper. Furthermore, visualization of internalized pHrodo bioparticles are observed in Fig. S2 (in supplementary information).

##### THP-1 cells adhesion and survival after 24 h copper release

To create conditions that mimic the dynamic fluid exchange of in vivo conditions, a 24 h leaching process of the material into RPMI was conducted prior to cell seeding. THP-1 cells were then seeded on pre-leached materials and cultured for 24 h, following the methods described in section 2.3.2 for evaluation of cell adhesion and viability, and ICP-OES as described in section 2.1.2. for quantification of released Cu ions.

##### Immunocytochemistry of CD206 and CD86

After the culturing procedure described in section 2.3.2, THP-1 cells adhered to the materials were fixed for 15 min with 4% formaldehyde at RT and washed twice with Tris-buffered saline-Tween (TBS-T, Sigma-Aldrich, St Louis, USA) at RT for 10 min. Cells were then blocked with 4% bovine serum albumin (BSA) (Sigma-Aldrich, St Louis, USA) in TBS-T for 30 min and incubated overnight at 4 °C with the following antibodies diluted (1:100) in 1% BSA in TBS-T: Anti-Mannose Receptor CD206 (Abcam (ab64693), Bristol, United Kingdom) and recombinant Anti-CD86 (Abcam (ab239075), Bristol, United Kingdom). CD206 is a marker for macrophage phenotype M2 and CD86 is a marker for M1. Cells were washed three times for 5 min with TBS-T. Next, cells were incubated for 2 h with the secondary antibody (Alexa Fluor-488-conjugated anti-rabbit IgG antibody (Abcam (ab150077), Bristol, United Kingdom)) at 1:500 dilution. Cells were then washed three times with PBS. Fluorescence was measured at 485/520 nm in a microplate reader. Next, cells adhered to the materials were stained with phalloidin (ActinRed™ 555 ReadyProbes™ Reagent (Rhodamine phalloidin), Invitrogen, Waltham, MA, USA), that selectively binds to F-actin, as a marker of total adhered cells. Fluorescence was measured at 544/590 nm. The fluorescence intensity (FI) from M1 and M2 markers was normalized to F-actin FI to account for potential cytotoxic effects of the materials. THP-1 macrophages previously polarized to M1 using 20 ng/mL of lipopolysaccharide (LPS; Invitrogen, Waltham, MA, USA) or to M2 using 25 ng/mL of IL-4/ IL-13 (PeproTech, Rocky Hill, NJ, USA) were used as positive controls.

##### Enzyme-linked immunosorbent assay-based (ELISA) detection of TNF-α and MRC1

After following the culturing procedure described in section 2.3.2, the THP-1 cell culture supernatant was collected for protein secretion analysis using the ELISA kits for human macrophage Mannose Receptor 1 ((MRC1); (EH329RBX10), Invitrogen, Waltham, MA, USA) and Tumor Necrosis Factor-alpha ((TNF-α); (DTA00C), R&D Systems, Minneapolis, MN, USA). The collected media were centrifuged at 300 *g* for 5 min, and the supernatants were aliquoted and stored at −80 °C until analysis according to the manufacturer’s instructions. In brief, undiluted samples were incubated with primary antibodies and washed followed by the addition of secondary antibody for immunoreactivity detection using horseradish peroxidase. Absorption was measured at 450 nm with wavelength correction. Protein concentrations were calculated by linear regression with a standard curve in the range of 0–2000 pg/mL for TNF-α and 0–100 ng/mL for MRC1. THP-1 macrophages previously polarized to M1 using 20 ng/mL of lipopolysaccharide (LPS; Invitrogen, Waltham, MA, USA) or to M2 using 25 ng/mL of IL-4/IL-13 (PeproTech, Rocky Hill, NJ, USA) were used as a positive control.

##### Scanning electron microscopy (SEM) of surface-adhered THP-1 cells and *S. aureus* bioparticles

After following the culturing procedure described in section 2.3.2, The morphology of THP-1 cells on the Control-Ti and Cu-Ep-Ti surfaces ± pHrodo™ red *S. aureus* bioparticles were visualized by SEM. Samples were washed with Hank’s Balanced Salt Solution (HBSS) (Thermo Fisher Scientific, Roskilde, Denmark) and fixed in 4% formaldehyde at 4 °C overnight to crosslink intracellular structures. Next, samples were rinsed with 0.15 M sodium cacodylate buffer (Sigma-Aldrich, St Louis, USA) and postfixed with 1% osmium tetroxide (Sigma-Aldrich, St Louis, USA) for 2 h on ice. The procedure continued as described for the SEM analysis of surface-adhered *S. aureus* biofilms in section 2.3.1.2.

### Indirect evaluation of material surfaces on *S. aureus* and THP-1 macrophages

“Indirect” refers to the seeding and growth of *S. aureus*, differentiated THP-1 macrophages or hFOB 1.19 osteoblasts in a growth medium conditioned by the presence of Cu-Ep-Ti or Control-Ti. To prepare conditioned media, the material discs were incubated statically in RPMI or TSBg under standard conditions for a defined period. All indirect evaluation experiments were performed with 3 independent biological experiments (*n* = 3) and 2 technical replicates.

#### Bactericidal properties of material-conditioned media against *S. aureus*

To evaluate the bactericidal properties of the released ions from Cu-Ep-Ti, discs were incubated statically in a 24-well microtiter plate at 37 °C for 24 h in 1 mL of either RPMI or TSBg. In addition to the Control-Ti-conditioned media, “aged” media wells were prepared following the same procedure but without the presence of any material disc – these along with “fresh” media were included as controls. The Cu-Ep-Ti and Control-Ti conditioned media are herein referred to as “material-conditioned media”. Five hundred microliters of conditioned media were transferred to a sterile 48-well plate (Thermo Fisher Scientific, USA). Following the bacterial culture preparation from section 2.2.1, a 10^6^ CFU/mL inoculum was added into the conditioned media to achieve a final concentration of 10^5^ CFU/mL and incubated at 37 °C, under shaking (125 rpm) for 24 h. Viable colony counting was performed from each well according to the planktonic procedure described above (2.3.1.1).

##### *S. aureus* intracellular reactive oxygen species (ROS) generation in material-conditioned media

An *S. aureus* inoculum (10^5^ CFU/mL) in RPMI prepared as described above was incubated at 37 °C under shaking (125 rpm) for 4 h. The culture was centrifuged in a benchtop centrifuge (Hettich, Kirchlengern, Germany) at 18 620 *ɡ*, for 8 mins and resuspended in 24 h Cu-Ep-Ti or Control-Ti conditioned media. These were then incubated for a further 30 min at 37 °C under shaking (125 rpm). Following this, CellRox Green Reagent^TM^ (Thermo Fisher Scientific, Carlsbad CA, USA) was added for a final concentration of 5 µM and incubated for 30 min at 37 °C under shaking (125 rpm). Bacterial cells were pelleted by centrifugation at 18 620 *g* and rinsed with PBS before being transferred to a black 96-well plate (Corning, New York, USA). FI was measured at 485/520 nm in a microplate reader.

#### THP-1 macrophage and hFOB 1.19 osteoblasts response to material-conditioned media

To assess the potential cytotoxicity of copper ions released from the Cu-Ep-Ti, materials in 1 mL RPMI 1640 were incubated statically at 37 °C for 4 h (conditioned media 1 (CM 1)). Subsequently, the CM 1 was collected, and new media were added and incubated for another 4 h period (8 h in total) (CM 2). This process was repeated, collecting the CM 2, replacing with fresh media, and collecting after 16 additional hours (24 h in total) (CM 3), followed by one final collection and media exchange for 24 h more (48 h in total) (CM 4).

Human fetal osteoblasts (hFOB 1.19, ATCC CRL-11372, Manassas, VA, USA) were cultured as described in Supplementary Methods. Cells were detached using 0.25% trypsin-EDTA for 5 min at 37 °C. The cell concentration was then adjusted to 60,000 cells/mL and seeded into a 96-well culture plate (Thermo Fisher Scientific, Waltham, MA, USA) for subsequent experiments. The osteoblasts were treated with different conditioned media (CM1, CM2, CM3, and CM4) for 24 h. After the treatment period, cells were detached using trypsin and centrifuged at 200 g for 3 min. The cell pellet was resuspended in 1 mL of RPMI medium, and 150 µL was used to quantify cell viability using the NucleoCounter®, which determined both the total cell count and the number of non-viable cells as previously described.

For differentiation towards macrophage-like cells, THP-1 cells were grown in T75 flasks and stimulated with 10 ng/mL PMA for 48 h followed by 24 h resting. To confirm successful differentiation, the expression of CD14 was evaluated by immunocytochemistry following the previously described protocol using CD14 monoclonal antibody conjugated Alexa Fluor 488 (Invitrogen (53-0149-42), Waltham, USA) (Fig. S3, in supplementary information). Adhered cells were then detached by treatment with trypsin-EDTA (0.25%) for 5 min at 37 °C and the cell concentration was adjusted to 100,000 cells per mL in a cell culture treated 96-well plate (Thermo Fisher Scientific, Waltham, USA) for the subsequent experiments. After 24 h growth in all the conditioned media, cells were treated with trypsin and centrifuged at 200 *g* for 3 min. The supernatant was used to quantify Cu ions released from each sample using ICP-OES as described. Cells were resuspended in 1 mL of RPMI. A 150 µL volume was used to quantify viability by NucleoCounter® to determine total cell count and non-viable cells as described above. In addition, 100 µL of cell suspension was transferred to a sterile 96-well plate containing 50 µL pHrodo™ red *S. aureus* bioparticles suspended in Live Cell Imaging Solution and incubated for 2 h at 37 °C. Fluorescence intensity was measured as described. Further, to evaluate cell recovery, 700 µL of cell suspension was incubated statically at 37 °C for a subsequent 24 h and cell viability was quantified using NucleoCounter®.

##### Immunocytochemistry of CD206 and CD86

THP-1 cells were differentiated as indicated in section 2.4.2. After 24 h growth in each of the conditioned media, cells were fixed for 15 min with 4% formaldehyde at RT and immunostaining was performed as described previously.

##### Enzyme-linked immunosorbent assay-based detection of TNF-α

THP-1 cells were differentiated as indicated in section 2.4.2. After 24 h growth in each of the conditioned media, the cell culture supernatant was collected for protein secretion analysis using a TNF-α ELISA kit as described previously.

### Statistics

Statistical analyses were performed in GraphPad Prism 9.5.1 (GraphPad, San Diego, CA, USA). For the evaluation of material characterization by contact angle, statistical significance was assessed using unpaired Student t-tests. For surface roughness, statistical significance was assessed using the Mann-Whitney test. For ion release, statistical significance was assessed using two-way ANOVA for multiple comparisons with Bonferroni’s *post-hoc* test. For evaluation of direct bacterial viability, prolonged copper ion exposure, *S. aureus* intracellular ROS production, direct and indirect THP-1 cell viability, direct and indirect phagocytosis, direct cell adhesion post-leaching and indirect recovery, statistical significance was assessed using unpaired Student t-test. For the indirect bacterial viability, an ordinary one-way ANOVA for multiple comparisons with Dunnett’s *post-hoc* test was applied. For direct and indirect ICC and ELISA one-way ANOVA for multiple comparisons with Tukey *post-hoc* test was applied. For all statistical analyses, *p* < 0.05 was considered statistically significant.

## Results

### Electrochemical deposition of Cu increases hydrophobicity and surface roughness of Cu-Ep-Ti

**Fig. 1:**
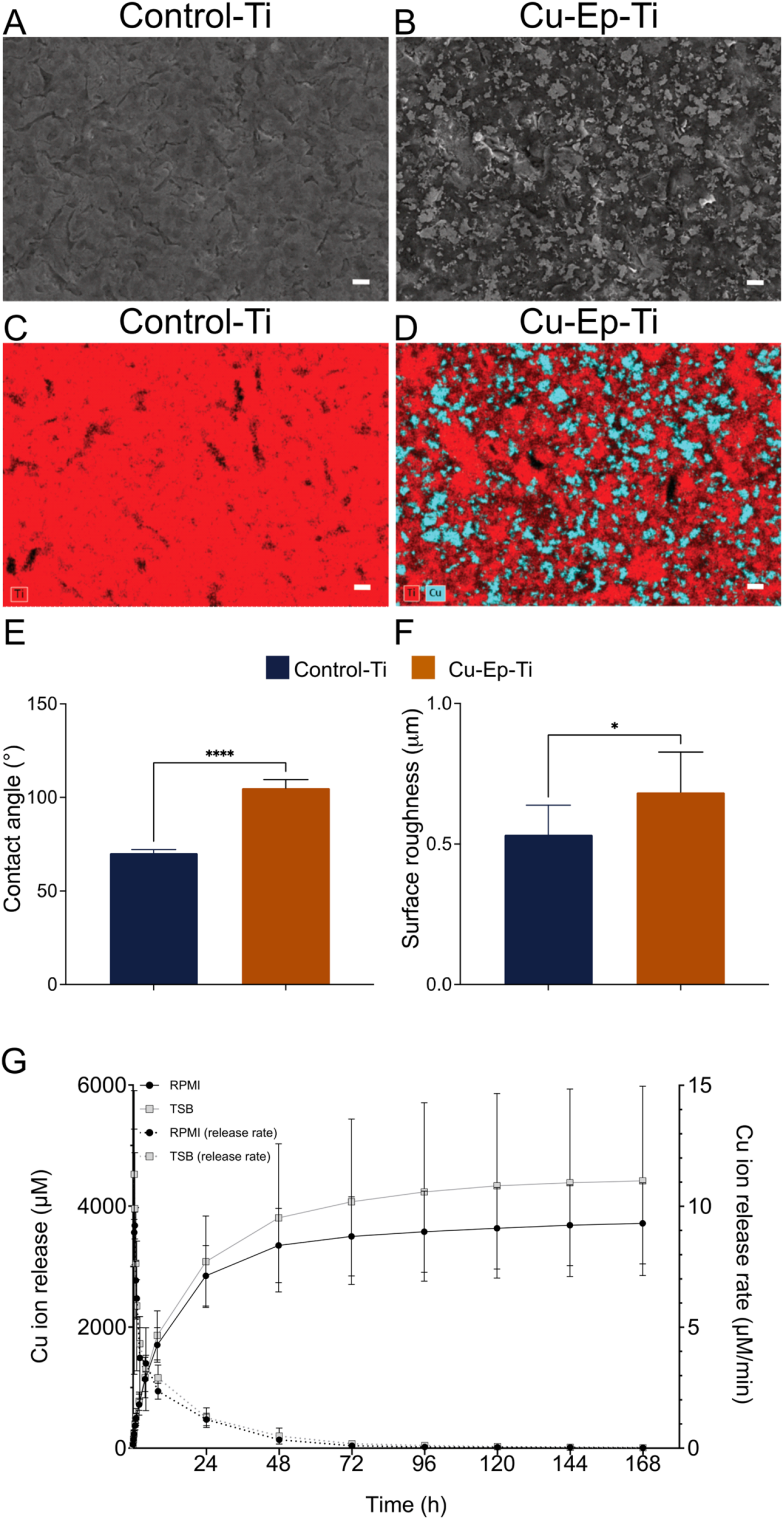
Characterization of the material surface properties. **A** B(S-SEM image of Control-Ti and (**B**) Cu-Ep-Ti surface. **C** EDX map of Control-Ti and (**D**) Cu-Ep-Ti showing Ti (red) and Cu (blue) presence and distribution on the material surface. **E** Contact angle of Control-Ti and the Cu-Ep-Ti. **F** Surface roughness of Control-Ti and Cu-Ep-Ti. **G** Cumulative Cu ion release and ion release rate from Cu-Ep-Ti, over 7 days (168 h) in RPMI and TSBg. Each bar represents the mean from (**E**), *n* = 4; **F** Control-Ti *n* = 5, Cu-Ep-Ti *n* = 9; (**G**), *n* = 6 Each bar graph represents the mea*n* ± SD. *≤ 0.05, ****P ≤ 0.0001, statistical significance tested against the Control-Ti using for (**E**) unpaired Students T-test, **F** Mann-Whitney test and (**G**) two-way ANOVA. Scale bar: (**A**–**D**) 10 μm

The presence and distribution of copper after electrodeposition was investigated with BSE-SEM and SEM-EDX. Micrographs of the Control-Ti and Cu-Ep-Ti show microscale roughness induced by the Blasting with glass pearls (diameter 40–70 µM) (Fig. 1A, B). The BSE-SEM micrographs of the Cu-Ep-Ti also show a heterogenous distribution of island-like copper deposits. These were confirmed as copper-islands by EDX mapping (Fig. 1C, D). In contrast, the Control-Ti solely shows the presence of Ti (Fig. 1C).

The wettability of the surfaces was determined through water contact angle measurements. Control-Ti exhibited hydrophilic properties, as indicated by a recorded contact angle of 70.23° (Fig. 1E). Conversely, the Cu-Ep-Ti showed significantly higher hydrophobicity, with an average contact angle of 105.05°, 35° higher than that of the Control-Ti.

Roughness profiles showed that the Cu-Ep-Ti surface (Fig. 1F) exhibited an average roughness of 0.68 µM (Pa), which was 0.15 μM rougher compared to the Control-Ti (0.53 µM (Pa)).

Further, copper ion release from Cu-Ep-Ti was quantified using ICP-OES in both RPMI and TSB. No statistically significant differences were observed between the release of copper ions in RPMI or TSB. The highest amount of copper ion release was measured between 8 and 24 h (1140 μM), cumulatively, however, the concentration begins to plateau after 48 h. Moreover, after 7 days of immersion, copper ions were still detectable, albeit in very small quantities (Fig. 1G).

These results show that copper electrodeposition increases hydrophobicity and surface roughness, and the release of ions is not medium-dependent, peaks at 24 h, and plateaus after 48 h.

### Evaluation of the direct *S. aureus* and THP-1 cell adhesion to the materials

#### Direct exposure to Cu-Ep-Ti reduces *S. aureus* ATCC 25923 viability in TSBg, is strongly bactericidal in RPMI and disrupts biofilm morphology

**Fig. 2:**
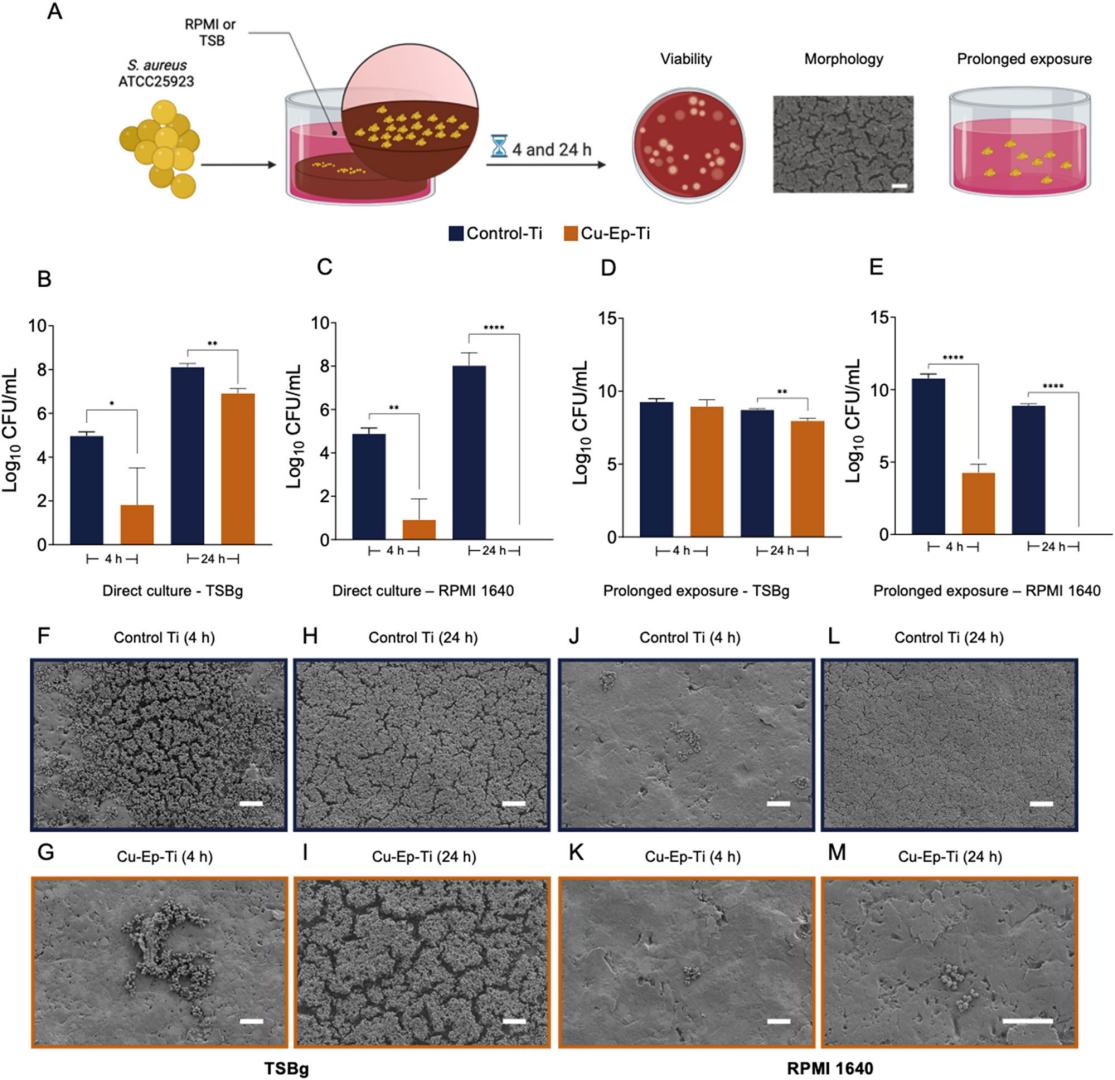
Direct adhesion and viability of *Staphylococcus aureus* ATCC 25923 on Control-Ti and Cu-Ep-Ti. **A** The experimental workflow undertaken to characterize the effect of the Cu-Ep-Ti specimens on *S. aureus* ATCC 25923 viability and morphology. **B**, **C** The direct effect of Cu-Ep-Ti on *S. aureus* ATCC 25923 viability in TSBg and in RPMI, respectively. **D**, **E** The effect of a further 24 h incubation with Cu-Ep-Ti sample-conditioned media on planktonic *S. aureus* viability after 4 or 24 h interaction with Cu-Ep-Ti or Control-Ti in TSBg or RPMI respectively. **F**–**I** Scanning electron micrographs of *S. aureus* ATCC 25923 morphology grown on top of the Control-Ti and Cu-Ep-Ti after 4 and 24 h in TSBg or (**J**–**L**) RPMI 1640. Each bar graph represents the mean from three independent experiments ± SD. *P ≤ 0.05, **P ≤ 0.01, ****P ≤ 0.0001, statistically significant compared against the Control-Ti using an unpaired Student T-test. Scale bars (**F**–**M** = 10 µM)

To understand the effect of Cu-Ep-Ti on bacterial viability and morphology, *S. aureus* ATCC 25923 was cultured directly on the material surface in TSBg or RPMI (Fig. 2A). After 4 h in both media conditions, the presence of copper resulted in a significant decrease in *S. aureus* viability (Fig. 2B, C) when compared to the Control-Ti. The bactericidal effect compared to the Control-Ti was slightly more pronounced in the RPMI than in TSBg (3.96 log_10_ CFU/mL (99.99%) *vs* 3.16 log_10_ CFU/mL (99.93%)).

This reduction in *S. aureus* viability at the material surface was also present at 24 h in both media. In TSBg, viability was significantly lower with a 1.2 log_10_ CFU/mL (93.7%) reduction observed in *S. aureus* adhered to Cu-Ep-Ti compared to the Control-Ti (Fig. 2B). However, similarly to the effects observed after 4 h, the bactericidal properties of the Cu-Ep-Ti were more pronounced in the RPMI condition. After 24 h, a complete bactericidal effect with no viable *S. aureus* adhered to the Cu-Ep-Ti material surface was observed (a reduction of 8.02 log_10_ CFU/mL (100%)) (Fig. 2C).

Following the 4 or 24 h direct adhesion with the Cu-Ep-Ti or the Control-Ti surfaces, *S. aureus* from the planktonic phase was transferred to a clean 24-well plate and re-incubated at 37 °C for a further 24 h to assess if bacterial fitness was permanently affected. In TSBg, the decreased viability observed after brief interactions (4 h) between *S. aureus* and the Cu-Ep-Ti surface (Fig. 2B) could be recovered from within the following 24 h (Fig. 2D) and the viability of the planktonic bacterial cells was comparable between Cu-Ep-Ti and Control-Ti. However, extended interactions (24 h) with the Cu-Ep-Ti (Fig. 2B) had a prolonged antimicrobial effect, with a significant 0.77 log_10_ CFU/mL (82%) reduction compared to Control-Ti after a further 24 h incubation (Fig. 2D). In RPMI, a significant prolonged effect on *S. aureus* bacterial fitness was observed after both 4- and 24 h of direct interaction with planktonic *S. aureus* cells. After 4 h, *S. aureus* viability was significantly lower (6.5 log_10_ CFU/mL (99.99%)) than Control-Ti, and no viable bacteria were recovered after prolonged incubation when planktonic bacteria interacted with Cu-Ep-Ti for 24 h (Fig. 2E) leading to a complete eradication.

In addition, the effect of the Cu-Ep-Ti material on biofilm morphology was qualitatively investigated using SEM. After 4 h of growth in TSBg, much of the material surface remained uncolonized, however, clusters of *S. aureus* cells were frequently observed, distributed across the Control-Ti surface with biofilms beginning to form (Fig. 2F). Comparatively, considerably fewer *S. aureus* cells were adhered to the Cu-Ep-Ti surface (Fig. 2G). Small aggregates of cells were observed dispersed across the surface, but markedly less frequently and with less cell density (Fig. 2G) compared to the aggregates formed on the Control-Ti (Fig. 2F). After 24 h of growth in TSBg, a significant biofilm was formed on the Control-Ti surface, coverage was dense and thick across almost the entire surface, with extracellular polymeric substance (EPS) production observed throughout the biofilm (Fig. 2H). A significant biofilm formation was also observable covering the Cu-Ep-Ti surface after 24 h in TSBg (Fig. 2I). However, unlike the Control-Ti, much of the underlying Cu-Ep-Ti surface remained uncolonized, though great heterogeneity in Z-coverage was observed, with clear zones developing into large multi-layered aggregates of biofilm. Notably, the channels in between aggregates were visibly larger in the surface of Cu-Ep-Ti (Fig. 2I) compared to Control-Ti (Fig. 2H).

After 4 h of growth in RPMI, the amount of *S. aureus* cells adhered to the material surface was sparse, with infrequently identified isolated clusters of cells on both Cu-Ep-Ti and Control-Ti materials (Fig. 2J, K). However, after 24 h, an extensive biofilm covered the entire Control-Ti surface (Fig. 2L), the biofilm thickness was visibly lower than that of the 24 h TSBg condition (Fig. 2H, Fig. S4), and cells within the biofilm exhibited a collapsed morphology (Fig. 2L, Fig. S4). Contrastingly, after 24 h of exposure to the Cu-Ep-Ti in RPMI, the quantity of *S. aureus* cells colonizing the material surface remained similar to that of the 4 h, with only small clusters of cells observed (Fig. 2M). Morphologically, the *S. aureus* cells were notably more wrinkled, with ruptured and burst cells being more commonly observed on Cu-Ep-Ti compared to on Control-Ti.

In conclusion, these results show that the presence of electrochemically deposited copper significantly reduces bacterial viability after 4 h and 24 h in both tested media. Moreover, the Cu-Ep-Ti material elicited a less developed biofilm morphology, decreasing biofilm density in TSBg, and in congruence with the viability counting, drastically inhibiting biofilm formation in RPMI.

#### Direct contact with Cu-Ep-Ti reduces THP-1 macrophage viability, and increases phagocytosis while also increasing the production of CD206/MRC1

**Fig. 3:**
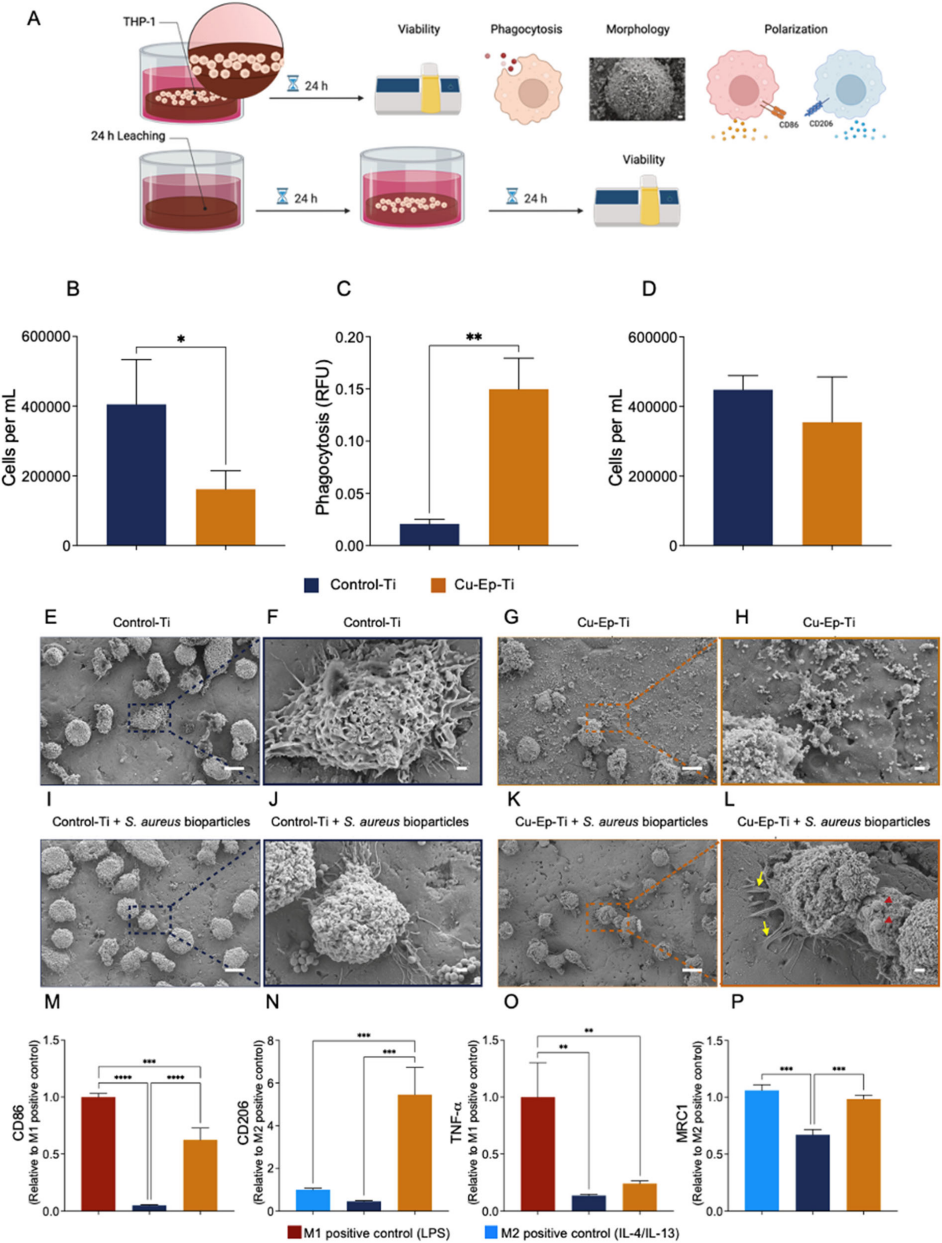
Direct adhesion, viability and polarization of THP-1 macrophages on Control Ti and Cu-Ep-Ti materials. **A** The experimental workflow undertaken to characterize the direct effect of the Control Ti and Cu-Ep-Ti materials on THP-1 macrophage-like behavior. **B** Number of THP-1 macrophage adhered to the surfaces after 24 h of direct contact with Control Ti and Cu-Ep-Ti materials. The released copper concentration from the same Cu-Ep-Ti samples was in average 1236 ± 459 μM. **C** 2 h phagocytosis of *S. aureus* bioparticles by THP-1 macrophages after 24 h of direct contact with Control Ti and Cu-Ep-Ti materials. **D** THP-1 macrophages adhered to 24 h pre-leached samples and further 24 h of direct contact with Control Ti and Cu-Ep-Ti. The released copper concentration from the same Cu-Ep-Ti samples was in average 169 ± 48 μM. **E**–**L** Scanning electron micrographs of THP-1 macrophages grown on either Control Ti (**E**, **F**, **J**, **I**) or Cu-Ep-Ti (**G**, **H**, **K**, **L**) samples with or without pHrodo *S. aureus* bioparticles. Relative fluorescence of (**M**) M1 marker (CD86) and (**N**) M2 marker (CD206) after 24 h of direct contact with Control Ti and Cu-Ep-Ti. Relative protein secretion of (**O**) TNF-α and (**P**) MRC1 after 24 h of direct contact with Control Ti and Cu-Ep-Ti. Pseudocolored versions of (**J** and **L**) are available Fig. S5 in supplementary information. Each bar graph represents the mean from three independent experiments (*n* = 3) ± SD. *P ≤ 0.05, **P ≤ 0.01, ***P ≤ 0.001 ****P ≤ 0.0001, statistical significance tested against the Control-Ti using for (**B**–**D**) unpaired Students T-test and (**M**–**P**) One-way ANOVA with Tukey *post-hoc* test. Scale bars (**E**–**G**–**I**–**K**) 10 µM, (**F**–**H**–**J**–**L**) 1 µM. Red arrows point to *S. aureus* bioparticles and yellow arrows point to THP-1 cell podosomes

To understand the cellular response (viability, phagocytosis, morphology, and polarization) to the Cu-Ep-Ti, THP-1 cells were cultured on the material surface (Fig. 3A). Unsurprisingly, a reduction in the viability of THP-1 cells was observed after 24 h when compared to the Control-Ti (Fig. 3B). In parallel, the copper ions released from the samples were quantified after 24 h. The average total copper release was 1236 μM (Fig. 3B).

Furthermore, the ability of the THP-1 cells to eradicate bacteria through phagocytosis was investigated by quantifying the fluorogenic response of pHrodo™ red *S. aureus* bioparticles during phagolysosome acidification. Viable THP-1 cells cultured on top of the Cu-Ep-Ti exhibited significantly increased phagocytosis of *S. aureus* bioparticles compared to those adhered to the Control-Ti material surface (Fig. 3C).

To mimic the dynamic fluid exchange in the body, a 24 h leaching of the material was conducted prior to seeding THP-1 cells for an additional 24 h to determine if the cells were capable of populating the surface. Importantly, no significant disparity in cytotoxicity was detected between the Control-Ti and the Cu-Ep-Ti material (Fig. 3D). In parallel, the copper ions released were quantified following the experiment in which the average copper release was 142 μM (Fig. 3D).

Further, the effect of Cu-Ep-Ti on THP-1 cell morphology was investigated qualitatively using secondary electron SEM after 24 h adhesion. On both materials, THP-1 macrophages were found randomly distributed across the surfaces (Fig. 3E, L). Cells cultured on the Control-Ti surfaces presented a polygonal morphology with normal size and intact membranes. Cytoplasmic extensions in several directions were observed, indicating cells were able to establish interactions with each other through podosomes (Fig. 3E, F). In contrast, cells adhered to Cu-Ep-Ti surfaces appeared mostly round-shaped with a small number of short protrusions and in some cases fragmented cytoskeletons. Additionally, several vesicle-like particles measuring approximately 50–180 nm in diameter were observed surrounding the THP-1 cells (Fig. 3G, H).

In addition, the morphology of THP-1 cells after 24 h adhesion with the surfaces was investigated after the addition of *S. aureus* bioparticles. Interestingly, the morphology remained unchanged when compared to samples without bioparticles. However, a higher concentration of bioparticles was observed outside of the THP-1 cells and on the surface of the Control-Ti material (Fig. 3I, J). In contrast, only a few bacterial bioparticles were observed on the Cu-Ep-Ti surface or associated to the membranes of THP-1 cells (Fig. 3K, L).

To evaluate whether direct contact between the THP-1 cells and Cu-Ep-Ti could influence their polarization into classically activated M1 macrophages or alternatively activated M2 macrophages, the expression levels of mannose receptor CD206 (M2 marker) and CD86 (M1 marker) were quantified by immunocytochemistry after 24 h of direct contact. Compared with the M1 positive control, the relative expression of CD86 was reduced to 0.6 in Cu-Ep-Ti while Control Ti was reduced to 0.05 (Fig. 3M). The relative expression of CD206 in Cu-Ep-Ti was 5-fold greater than the M2 positive control, while the expression in Control-Ti was reduced to 0.4 when compared with M2 positive control (Fig. 3N). To better understand the effect of the Cu-Ep-Ti on the THP-1 cell behavior, the levels of TNF-α (M1 associated) and MRC1 (M2 associated) protein secretion, both with a central role in inflammation were quantified. Compared with the M1 positive control, the relative secretion of TNF-α by THP-1 cells cultured on Cu-Ep-Ti was decreased to 0.24 while on Control Ti it was lower at 0.13 (Fig. 3O). Compared with the M2 positive control, the relative secretion of MRC1 by THP-1 cells adhered to the Cu-Ep-Ti was 0.98 while in the Control Ti group, expression was lower at 0.67 when compared to the positive controls (Fig. 3P).

In summary, these results show that Cu-Ep-Ti reduces the viability of THP-1 cells after 24 h direct contact. However, if samples are leached or undergo dynamic fluid exchange, THP-1 cells can populate the surfaces and the viability is comparable to that of the Control-Ti. Cu-Ep-Ti materials were also found to enhance THP-1 cell phagocytosis and in line with this an increased expression of the M2 markers (CD206 and MRC1) was observed in comparison to Control-Ti.

### Indirect evaluation

#### The Cu-Ep-Ti surface releases bactericidal concentrations of copper ions and induces intracellular ROS in *S. aureus*

**Fig. 4:**
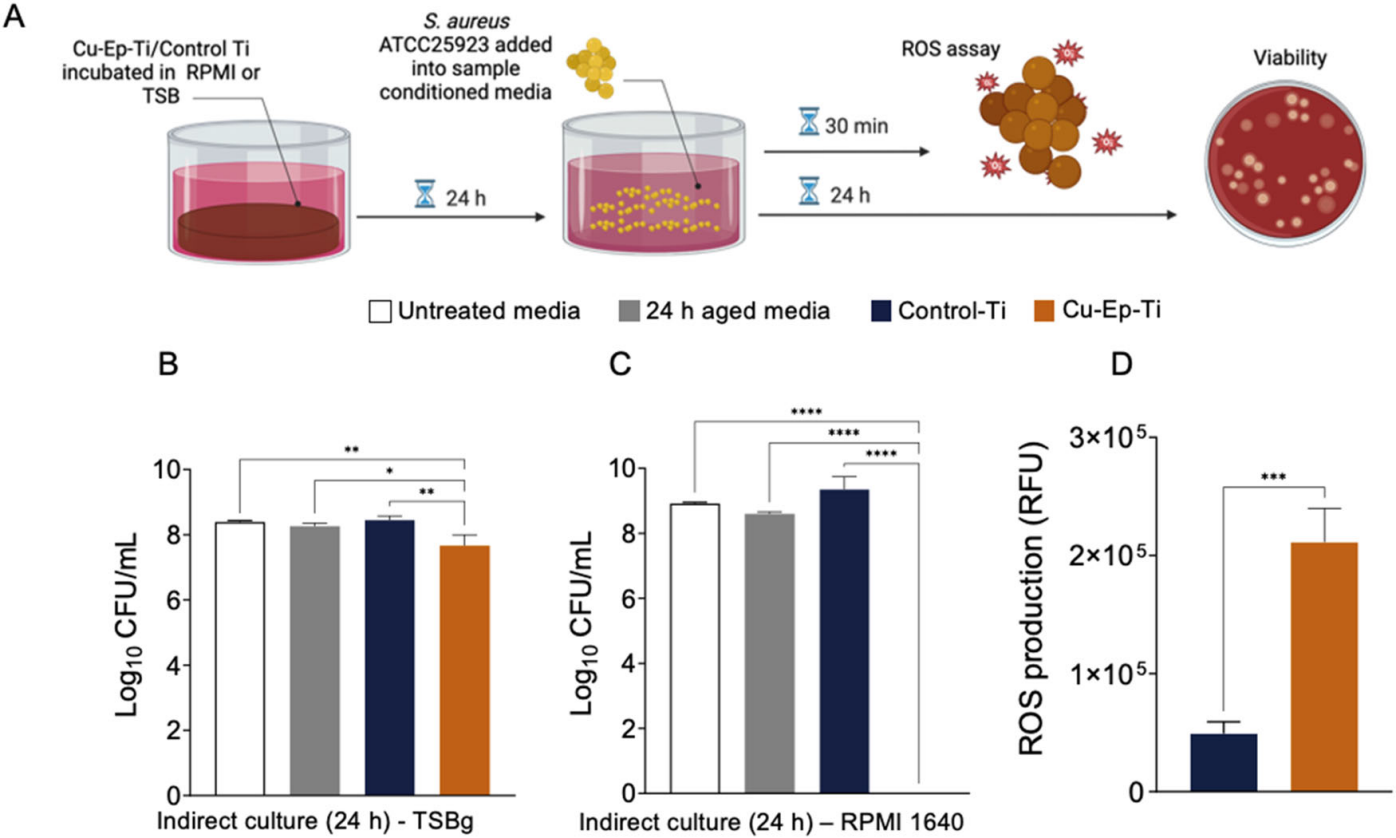
Indirect interactions *–* Viability and stress response of *S. aureus* after exposure to Control-Ti and Cu-Ep-Ti material-conditioned media. **A** Experimental methodology to investigate the role of indirect culture with Cu-Ep-Ti material-conditioned media on the biological responses of *S. aureus* ATCC 25923. **B** The effect of 24 h Cu-Ep-Ti material-conditioned media on *S. aureus* ATCC 25923 viability compared to the Control-Ti, growth media, and “aged” growth media after 24 h interaction in TSBg or (**C**) RPMI 1640. **D** Intracellular reactive oxygen species (ROS) production by *S. aureus* ATCC 25923 after 30 min exposure to Cu-Ep-Ti sample-conditioned media compared to the Control-Ti. Each bar represents the mean from three independent experiments (*n* = 3) ± SD. *P ≤ 0.05, **P ≤ 0.01, ***P ≤ 0.001, ****P ≤ 0.0001, statistically significant compared against the untreated Control Titanium and/or growth media only conditions using (**B**, **C**) One-Way ANOVA with Dunnett’s *post-hoc* for multiple comparisons or (**D**) unpaired Student T-test

With the observation that direct inoculation of *S. aureus* onto the Cu-Ep-Ti surface resulted in a significant decrease in viability in both media conditions, the bactericidal properties of copper ions released into the surrounding media were investigated by inoculating 10^5^ CFU/mL *S. aureus* into material-conditioned TSBg or RPMI for 24 h (Fig. 4A).

We found that in TSBg, copper ions from Cu-Ep-Ti were released at a high enough concentration to produce a measurable bactericidal effect, with a 0.78 log_10_ CFU/mL (83.5%) reduction identified versus the Control-Ti (Fig. 4B). While this effect was statistically significant, similar to the direct culture method, culturing in TSBg quenched the bactericidal properties compared to the RPMI condition. In congruence with the direct culture, the bactericidal effect of copper released into RPMI was significantly more pronounced than that in TSBg, with no viable *S. aureus* recovered after 24 h (Fig. 4C). In contrast, the Control-Ti a total of 9.3 log_10_ CFU/mL of *S. aureus* was recovered.

Moreover, after having observed significant bactericidal properties after *S. aureus* exposure to the Cu-Ep-Ti surface and material-conditioned media, the generation of reactive oxygen (superoxide anion and hydroxyl radical) species (ROS) was assessed using a fluorescence-based ROS assay. After 30 min exposure to Cu-Ep-Ti-conditioned media, *S. aureus* produced significantly more intracellular ROS compared to when *S. aureus* was exposed to Control-Ti-conditioned media (Fig. 4D).

In summary, these results demonstrate that the concentration of copper ions released from the Cu-Ep-Ti surface is bactericidal and induces significant ROS generation.

#### Indirect contact with Cu-Ep-Ti reduces THP-1 macrophage viability, increases phagocytosis and modulates macrophage polarization

**Fig. 5:**
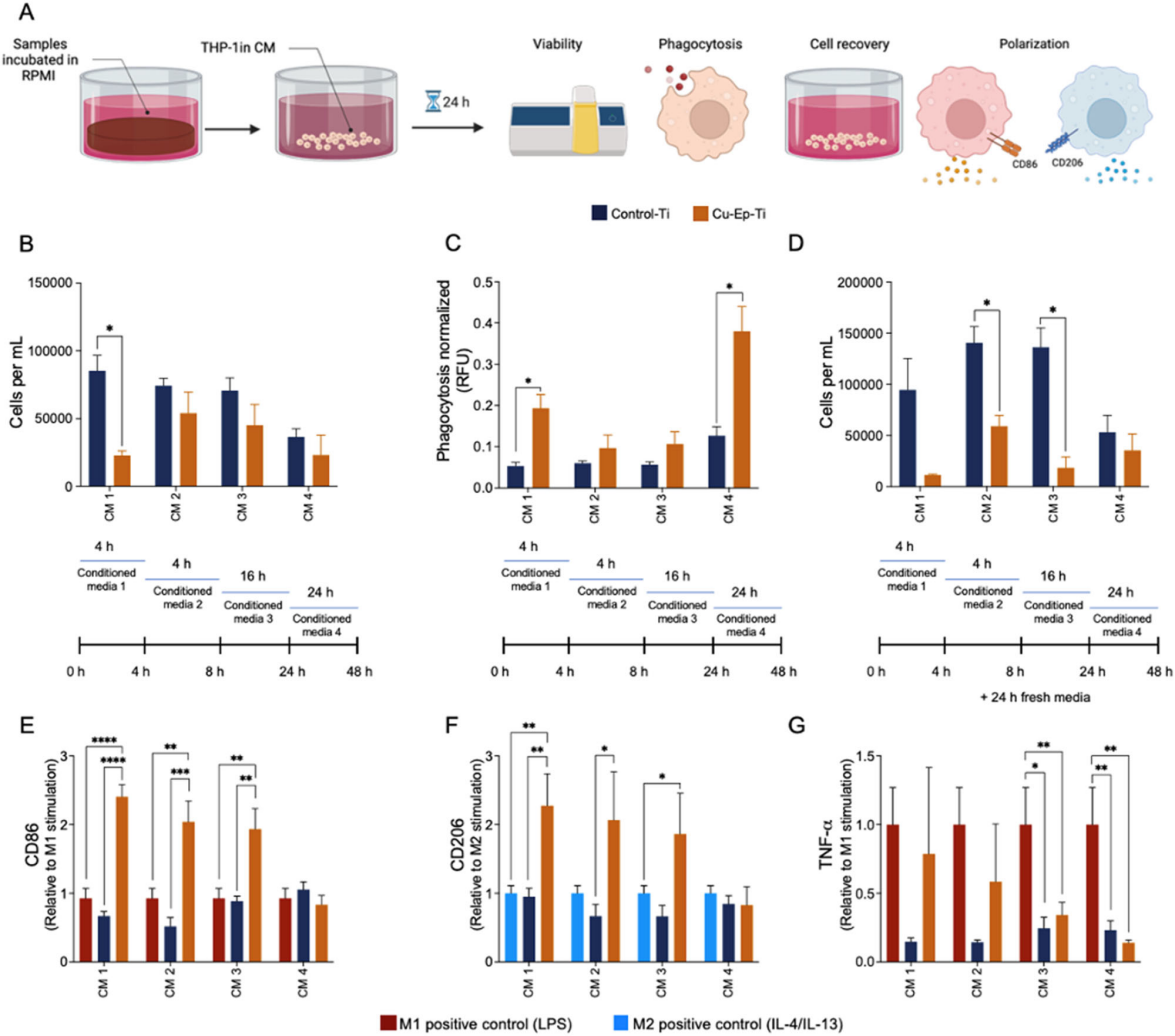
*Indirect contact* – behavior of THP-1 macrophages on Control-Ti and Cu-Ep-Ti material-conditioned samples. **A** The experimental workflow undertaken to characterize the effects of released ions (indirect effect) from the Control Ti and Cu-Ep-Ti materials on THP-1 macrophage-like behavior. **B** Number of viable THP-1 macrophages per mL. Copper concentration in conditioned media from Cu-Ep-Ti, CM1: 702 ± 96 μM; CM2: 402 ± 71 μM; CM3: 857 ± 370 μM; and CM4: 369 ± 168 μM. **C** Phagocytic activity of THP-1 macrophages after 24 h exposure to conditioned media. **D** THP-1 macrophage viability after 24 h recovery in fresh RPMI 1640. Relative fluorescence of (**E**) M1 marker (CD86) and (**F**) M2 marker (CD206) after 24 h of indirect contact with conditioned media. Relative protein secretion of (**G**) TNF-α after 24 h of indirect contact with conditioned media. Each bar represents the mean from three independent experiments (*n* = 3) ± SD. *P ≤ 0.05, **P ≤ 0.01, ***P ≤ 0.001, ****P ≤ 0.0001, statistically significant compared against the control Ti using (**B**–**D**) unpaired Student T-test and (**E**–**G**) One-way ANOVA with Tukey *post-hoc* test

To evaluate the behavior (cytotoxicity, phagocytosis, and polarization) of THP-1 macrophages exposed to copper ions released from the Cu-Ep-Ti, THP-1 macrophages were cultured for 24 h in each conditioned media (CM 1, CM 2, CM 3, and CM 4; Fig. 5A). A significant decrease in viable cells was observed in the presence of Cu-Ep-Ti CM 1. However, in CM 2, CM 3, and CM 4 the presence of copper ions did not lead to cytotoxicity when compared to Control-Ti (Fig. 5B). To understand the concentrations of copper that induced these effects, the total copper ions released were quantified from each conditioned media. For CM 1, the average copper content was 702 μM for CM 2, 422 μM for CM 3, 857 μM and for CM 4: 369 μM (Fig. 5B). Similarly, the effect of Cu-Ep-Ti conditioned media on the viability of human osteoblasts was also investigated. Here, the concentration of copper was more closely associated with cytotoxicity. In CM3 (8-24 h), where the highest amount of copper was released, a significant decrease in osteoblast viability was observed (Fig. S6, in supplementary information).

Further, we studied whether phagocytosis would be influenced by the copper ions released from the Cu-Ep-Ti. In agreement with the increase in phagocytosis after direct culture on Cu-Ep-Ti, we observed a significant increase in phagocytosis in the Cu-Ep-Ti-conditioned media CM 1 and CM 4 compared to Control-Ti (Fig. 5C). However, only a non-significant increasing trend was observed in CM 2 and CM 3 (Fig. 5C).

As the implant environment is dynamic, with a constant flow of nutrients and removal of waste, we investigated whether THP-1 macrophages could overcome any toxicity caused by the initial release of copper ions from the Cu-Ep-Ti material. THP-1 macrophages were treated with each of the sample-conditioned media for 24 h, after which fresh RPMI was added, and THP-1 cells were cultured for further 24 h. After treating THP-1 macrophages with Cu-Ep-Ti CM 1, no significant long-term reduction in viability occurred when compared to the Control-Ti (Fig. 5D). Moreover, when THP-1 cells were exposed to lower copper concentrations for longer periods of time (48 h Cu-Ep-Ti CM 4), the sustained effect of copper on cytotoxicity was negligible, and the viability of THP-1 macrophages were directly comparable to the Control-Ti (Fig. 5D). Conversely, the concentration of copper ions in the Cu-Ep-Ti CM 2 and CM 3 resulted in a significant sustained reduction in cell viability.

To evaluate whether the indirect contact of the THP-1 cells with Cu-Ep-Ti could influence macrophage polarization CD206 and CD86 markers were quantified by immunocytochemistry after 24 h growth in each conditioned media. Compared to the M1 positive control, the relative expression of CD86 was in CM1: significantly higher (2.4-fold) in Cu-Ep-Ti, reduced by 0.4 in Control-Ti (Fig. 5E); CM2: increased significantly (2-fold) in Cu-Ep-Ti and reduced by 0.5 in Control-Ti (Fig. 5E); CM3: was increased 1.9-fold in Cu-Ep-Ti while decreased by 0.2 in Control-Ti (Fig. 5E); CM4: expression of CD86 was equivalent to M1 positive control for both Cu-Ep-Ti and Control-Ti materials (Fig. 5E). In CM1, the relative expression of CD206 in Cu-Ep-Ti was significantly increased (2.3-fold) when compared to the M1 positive control whereas the Control Ti was slightly reduced (by 0.1) (Fig. 5F). In CM2, the relative expression of CD206 in Cu-Ep-Ti was also increased significantly (2-fold) when compared to the M1 positive control, while the Control Ti was reduced by 0.4 (Fig. 5F). In CM3, the relative expression of CD206 in Cu-Ep-Ti was again significantly greater than that of the M1 positive control (1.8-fold), while Control Ti was reduced by 0.34 (Fig. 5F). In CM4, similar to the CD86 expression, the relative expression of CD206 in Cu-Ep-Ti and Control Ti was not significantly changed when compared to the M1 positive control (0.2-fold reduction in both) (Fig. 5F). Moreover, to better understand the effect of the Cu-Ep-Ti conditioned media on the THP-1 macrophage inflammatory response, the levels of TNF-α (M1 associated) protein secretion were quantified. The relative secretion of TNF-α by THP-1 cells cultured in Cu-Ep-Ti (CM1) was reduced by 0.3 when compared with the M1 positive control, while on Control Ti, relative secretion was reduced by 0.9 (Fig. 5G). In CM2, the relative secretion of TNF-α was reduced by 0.5 in Cu-Ep-Ti while in Control Ti production was decreased by 0.9. In Cu-Ep-Ti CM3, the relative secretion of TNF-α was reduced by 0.7 similar to the Control Ti in which a reduction of 0.8 was observed. In CM4, relative TNF-α secretion was reduced by 0.9 in Cu-Ep-Ti and 0.8 in Control Ti compared to the M1 positive control (Fig. 5G).

In conclusion, these findings suggest that the copper ion concentration released from the Cu-Ep-Ti surface during the initial 4 h (CM1) reduces cell viability, however, the copper ion concentration released during the following 4 h (CM2), 16 h (CM3) and 24 h (CM4), result in a cell viability comparable to Control-Ti. Further, the phagocytic capacity of THP-1 cells is enhanced by the highest and lowest Cu ion concentrations (4 h/CM1 and 24 h/CM4) in Cu-Ep-Ti, however, a small increasing trend in phagocytosis was measured during the intermediate timepoints/conditioned media (4 h/CM2 and 16 h/CM3). Moreover, most of the Cu ion concentrations (CM1, CM2 and CM3) released by the Cu-Ep-Ti material, modulated the differentiation of macrophages towards both M1 and M2 phenotypes.

### Cu-Ep-Ti reduces the adherence and viability of an *S. aureus* clinical isolate from periprosthetic joint infection (PJI)

**Fig. 6:**
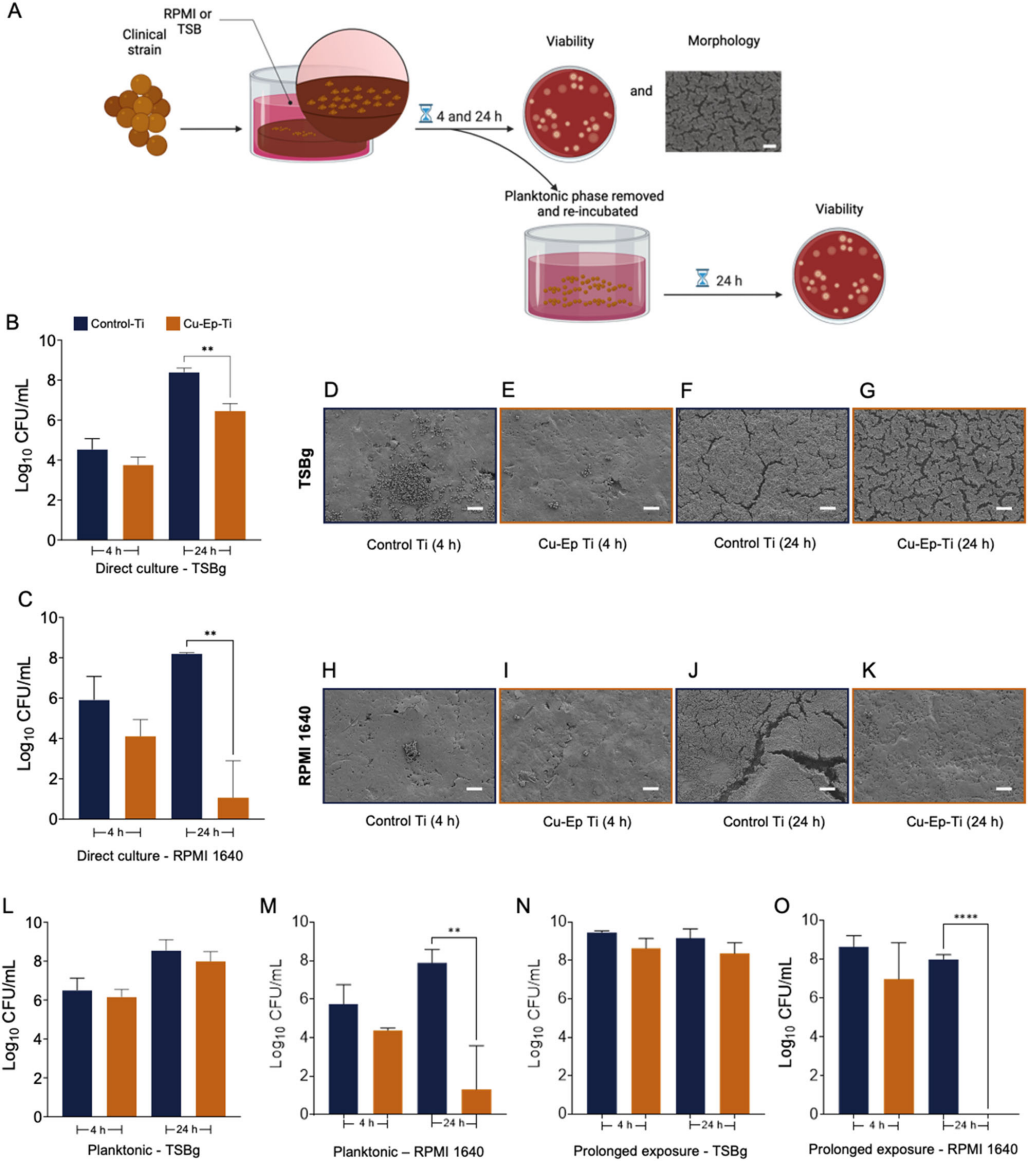
The effect of Cu-Ep-Ti on the adhesion, prolonged cytocompatibility, and morphology of a clinical *S. aureus* periprosthetic joint infection (PJI) isolate. **A** Experimental methodology to investigate the role of Cu-Ep-Ti interactions on the biological responses of a clinical *S. aureus* M.19.373 strain isolated from PJI. **B** The effect of Cu-Ep-Ti on the viability of the clinical *S. aureus* M.19.373 PJI isolate after 4- and 24 h direct culture in TSBg. **C** The effect of Cu-Ep-Ti on the viability of the clinical *S. aureus* PJI isolate after 4- and 24 h direct culture in RPMI 1640. **D**–**G** The respective representative scanning electron micrographs of *S. aureus* M.19.373 adhered to the Cu-Ep-Ti and Control-Ti surfaces after 4- and 24 h in TSBg. **H**–**K** The respective representative scanning electron micrographs of *S. aureus* M.19.373 adhered to the Cu-Ep-Ti and Control-Ti surfaces after 4- and 24 h in RPMI 1640. **L** Viable planktonic *S. aureus* M.19.373 cells following 4- or 24 h direct interaction in TSBg and (**M**) RPMI 1640. **N** The viability of a further 24 h incubation with copper of planktonic *S. aureus* M.19.373 after 4- or 24 h direct interaction with Cu-Ep-Ti or the Control-Ti in TSBg (**O**) or RPMI 1640. Each bar represents the mean from three independent experiments (*n* = 3) ± SD. **P ≤ 0.01, ****P ≤ 0.0001, statistically significant compared against the Control-T using unpaired Students T-test. Scale bars (**D**–**K**) = 10 µM

As the intended clinical application of the Cu-Ep-Ti biomaterial is an orthopedic implant device, *S. aureus* M19.373, a clinical strain isolated from a patient with periprosthetic joint infection (PJI), was also tested. This strain caused a relapse in the patient and is a strong biofilm producer. The Control-Ti and Cu-Ep-Ti were challenged with this *S. aureus* PJI strain in a similar manner to the lab strain (Fig. 6A). Here, unlike the lab strain, after 4 h of direct interaction, no bactericidal effect was observed in either TSBg or RPMI (Fig. 6B, C). However, as the interaction period continued to 24 h, a significant reduction (98.86%) in bacterial viability was observed (1.95 log_10_ CFU/mL) in TSBg (Fig. 6B, C) and as for the lab strain, direct interaction with the Cu-Ep-Ti for 24 h resulted in a pronounced bactericidal effect (99.99%) when *S. aureus* M19.373 was grown in RPMI (7.10 log_10_ CFU/mL) (Fig. 6C).

In addition, SEM was also performed to qualitatively observe the effect of Cu electroplating on the biofilm morphology of the M19.373 strain. After 4 h, cells from the *S. aureus* clinical PJI isolate formed small to medium communities arranged as monolayers in areas across the Control-Ti surface (Fig. 6D). In contrast, far fewer adhered *S. aureus* M19.373 cells were observed on the Cu-Ep-Ti with at most, clusters of around 20 cells distributed in areas across the surface (Fig. 6E). This reduction in the initial adhered cell number between the Cu-Ep-Ti compared with Control-Ti was also detected in RPMI (Fig. 6H, I). Clusters of greater cell density were more frequently observed on the Control-Ti than on Cu-Ep-Ti. Though on both material types at 4 h in RPMI *S. aureus* M19.373 adhered to the surface were sparse with significantly fewer cells and clusters in comparison to TSBg, likely reflecting the limited nutrition provided by RPMI when compared to TSBg (Fig. 6H, I). After 24 h biofilm formation in TSBg, the Control-Ti surface was completely covered by a dense thick biofilm with the underlying titanium no longer visible (Fig. 6F). Biofilm formation was also observed across the Cu-Ep-Ti surface at 24 h, with considerable EPS production visible in the biofilm, which was segregated by distinct channels (Fig. 6G). The biofilm formed on the Cu-Ep-Ti appeared comparatively less thick than that formed on the Control-Ti, as the material surface was visible through the biofilm channels. These biofilm channels were also wider and more numerous on the Cu-Ep-Ti surface, as also observed with the lab strain ATCC 25923. After 24 h in RPMI, a significant biofilm formation could also be observed on the Control-Ti surface with a heterogeneous biofilm morphology, ranging from cell monolayers to thick multi-layered biofilms (Fig. 6J). In contrast, almost no *S. aureus* M19.373 were present at the Cu-Ep-Ti surface after 24 h in RPMI (Fig. 6K), comparable to the adhesion level observed at 4 h (Fig. 6I). Small groups of cells could be observed, though these were few and infrequent. In general *S. aureus* M19.373 grown in RPMI exhibited slightly deflated cell morphologies.

Further, to assess whether Cu ions released from the Cu-Ep-Ti reached a bactericidal threshold, viable colony counting was performed from the planktonic *S. aureus* M19.373 cells that did not adhere to the materials. At the early time point (4 h) in both types of media, copper ions released from the Cu-Ep-Ti material did not generate a significant bactericidal effect against planktonic *S. aureus* M19.373 cells compared to Control-Ti (Fig. 6L, M). This was similar for 24 h in TSBg (Fig. 6L). However, after 24 h of interaction with the Cu-Ep-Ti in RPMI, the released ion concentration induced a significant bactericidal effect (99.99%) and a reduction in viability (6.57 log_10_ CFU/mL) of *S. aureus* M19.373 (Fig. 6M).

Compared to the lab strain, the recovery of the *S. aureus* PJI clinical isolate exhibited greater resilience to short-term exposure to released copper ions. After exposure of *S. aureus* M19.373 planktonic cells to the materials in TSBg for 4- or 24 h, and further 24 h re-incubation in fresh media, the viability of the Cu-Ep-Ti-treated planktonic cells was directly comparable to that of the Control-Ti (Fig. 6N). This was also observed in the 4 h Cu-Ep-Ti exposure condition in RPMI (Fig. 6O) where no difference in viability was observed. However, as for the lab strain, 24 h of direct interaction with the Cu-Ep-Ti specimen in and further 24 h re-incubation in RPMI, *S. aureus* M19.373 was completely eradicated, and no viable bacteria were counted (Fig. 6O). Taken together, these results demonstrate that a clinical PJI isolate of *S. aureus* exhibits slightly more recalcitrance to the Cu-Ep-Ti, however significant bactericidal effects are still observed after 24 h and this effect remains over prolonged incubation periods.

## Discussion

The antimicrobial properties of copper have been known for centuries, and much research has been undertaken to apply this property to almost all biomaterial types [26, 27]. More recently, the role of copper as an immunomodulatory element for biomaterial applications has also begun to be recognized [28, 29]. In the present study, we showed that electrochemically depositing metallic copper onto Ti6Al4V-ELI can generate both potent antimicrobial properties while simultaneously inducing a macrophage response. The observed immunostimulatory property could improve the local immune environment surrounding a newly emplaced implant.

To confirm the antimicrobial properties of the developed Cu-Ep-Ti material, disks were challenged with *Staphylococcus aureus* due to its role as one of the most significant pathogens associated with biomaterial-associated infections. Indeed, after 4- and 24 h in TSBg, a significant reduction in viability was observed – confirming the electrodeposition of copper conferred antimicrobial properties to the Ti6Al4V-ELI substrate. We also investigated if these antimicrobial properties would be sustained in cultured medium more reflective of the host’s physiological condition, therefore, experiments were also conducted in the common cell culture medium RPMI 1640. Interestingly, after 24 h, complete eradication of *S. aureus* (ATCC 25923) was observed. This increase in bactericidal activity was surprising, but not entirely unexpected. TSBg being designed for bacterial culture provides a far more nutritionally supportive environment for the proliferation of *S. aureus* when compared to RPMI. These differences in nutritional quality are most obviously highlighted in the SEM micrographs (Fig. 2F–M), where *S. aureus* adhesion and cell size are visibly reduced after growth in RPMI compared to TSBg – a known response to nutritional limitation/starvation [30]. The reduced toxicity observed after growth in TSBg could also be a function of the differing compositional complexity/richness of the media. Components of TSB such as peptone are known to complex free copper reducing their bioavailability and subsequent toxicity towards *S. aureus* [31].

Considering the future clinical application of this material, we also strove to assess the bactericidal properties of the Cu-Ep-Ti against a clinical *S. aureus* strain isolated from a periprosthetic joint infection. This strain showed slightly more resistance towards the bactericidal effects of the Cu-Ep-Ti after both 4- and 24 h, but significant reductions in viability remained after 24 h, similarly to the lab strain. These viability investigations were performed both in direct contact with the Cu-Ep-Ti surfaces as well as in media conditioned by the material samples. In both conditions, we have seen significant reductions in the viability of the challenged *S. aureus*, indicating that the bactericidal effects are likely due to copper ions released from the material surface, though contact killing is also likely for those cells that survive to interact with the material.

Strong biofilm formation in staphylococci causing periprosthetic joint infection has been significantly associated to an increased risk for recurrent infection in patients [32]. The present study showed a significant disruption of biofilm formation of the lab and clinical *S. aureus* strains when grown on the Cu-Ep-Ti, in both media conditions. As with the viability analyses, the observed effects were more significant in the minimal growth condition (RPMI 1640) – where biofilm formation was entirely prevented up to 24 h. In TSBg, biofilm formation was still observed, but in both lab and clinical strains biofilm morphology was visibly altered with wider nutrient and water diffusion channels present in both biofilms. This alteration in biofilm morphology may be related to the fact that copper stress has been found to decrease biofilm formation by repressing the expression of key biofilm regulators, such as the accessory gene regulator (*agr*) and *S. aureus* exoprotein expression locus (*sae*), along with reducing the expression of important biofilm proteins, such as the extracellular adherence protein (*eap*) [33]. The physical properties and nutrient gradients of the biofilm are known to prevent adequate diffusion of antibiotics into the core, while the altered metabolism of the enclosed cells increases their tolerance [34, 35]. It is therefore possible that this disruption of biofilm biomass and structure could contribute to improved therapeutic efficacy of procedures such as DAIR (debridement, antibiotics and implant retention) for periprosthetic joint infections, where the aim is to treat infection while retaining the implanted device.

Results from this study confirm bactericidal and antibiofilm effects of the Cu-Ep-Ti. The antimicrobial mechanisms of action of copper are numerous [33, 36–38]. Among them, we investigate whether exposure to copper ions released from the Cu-Ep-Ti could induce stress-related ROS production in *S. aureus*. Indeed, we observed a significant generation of endogenous ROS by *S. aureus* grown in Cu-Ep-Ti conditioned media compared to the equivalent grown in Control-Ti conditioned media. This is a known *S. aureus* stress response to the presence of excess/unmanageable concentrations of copper that cannot be overcome by the detoxifying action of the superoxide dismutase (SOD) [33]. The production of ROS is also a well-known antimicrobial mechanism exhibited by immune cells such as neutrophils and macrophages [39, 40]. A combination of endogenous stress-induced ROS production triggered by the Cu-Ep-Ti and exogenous ROS produced by local immune cells would present a strong double-barrelled antimicrobial environment against potential pathogens further improving in vivo infection resistance of the implanted device.

Ensuring a successful immune response to an implanted material is challenging, and while infection is the most life-threatening complication, aseptic failure due to immunological issues are a more common cause of orthopedic implant failure [41]. Importantly, the presence of copper has been shown to elicit several immunomodulatory effects that could improve the immune response to infection and to the implant [28, 29, 42, 43]. However, the Cu concentrations required to be bactericidal are also often cytotoxic towards host cells.

As maybe expected, here we observed a reduction in THP-1 macrophage cell viability after direct culture on the material surface and after early indirect culture. A reduction in human osteoblast viability was also observed after indirect culture (CM3) where copper concentrations were highest. Understanding the prolonged cytotoxic effects of an antimicrobial material is fundamental as in a clinical scenario, uncontrolled toxicity could jeopardize the desired function of the implanted device – especially where stable tissue integration is desired. However, it is important to acknowledge that these cytotoxicity investigations were performed under static conditions, whereas real in vivo conditions are often dynamic – allowing fresh nutrient supply and steady recruitment of new macrophages and osteoblasts. As such, the concentrations of copper to which cells are exposed in vivo, and the subsequent toxicity, would likely be lower. Indeed, when conditions were made more dynamic with 24 h leaching and media replacement, no significant cytotoxicity was observed in THP-1 macrophages with cell viability equivalent to the Control-Ti. Similarly, in sample-conditioned media experiments, only the early time point showed certain cytotoxicity. Interestingly, the concentration of Cu ions released from the Cu-Ep-Ti with macrophages present was ~900 μM lower than when macrophages were absent. It is possible that this reduction reflects the uptake and use of copper in macrophage metabolic and biosynthetic pathways. It is also relevant to consider however that surface adherent macrophages may acidify their local environment and increase Cu ion release. While this may improve the bactericidal properties and elevate phagocytosis, it could also contribute to the cytotoxicity of the Cu-Ep-Ti. Interestingly, despite the cytotoxicity identified, we observed a significant increase in the ability of the viable THP-1 macrophages, on the material surface and after treatment with released copper ions, to phagocytose staphylococcal bioparticles. Similar effects have been previously observed in vitro where the phagocytic capacity of murine macrophages was increased after treatment with zinc ions [44]. Regarding copper, some studies have reported that the presence of high concentrations of copper ions can also increase the efficacy of phagocytosis, improving the phagosomal degradation of ingested material. This is thought to be due to the elevated expression of ATP7A, CTR1, and ceruloplasmin, which facilitate the uptake and trafficking of copper into the cytoplasm and phagosome [45].

In this study, this increase in phagocytosis also corresponded with the observation that THP-1 cells cultured directly on Cu-Ep-Ti surfaces and exposed to released Cu ions differentiated towards M1 and M2-like macrophages, by the elevated expression and production of CD86 (M1) and CD206 (M2).

These activated macrophages play an important role in the immune response to implanted biomaterials. The currently understood dogma suggests that M1 (classically activated) macrophages are responsible for inflammatory processes and immune cell recruitment, whereas M2 (alternatively activated) are primarily responsible for coordinating anti-inflammatory processes and organizing reparative processes [46–48]. However, it is unlikely that the distinction is so binary in vivo. While both macrophage phenotypes are phagocytically active, alternatively activated macrophages tend to be more so than their classically activated counterparts [49, 50]. Therefore, the found increase in phagocytosis may be partly explained by the increased expression of CD206, as the primary function of this C-type lectin receptor is to mediate the internalization of pathogens, dead cells, and debris [51]. This increased differentiation towards alternatively activated M2-like macrophages may also be modulated by the topography of the Cu-Ep-Ti, as specific microroughness ( > 0.51 Ra) have been found to play a role in the differentiation of THP-1 cells towards M2-macrophage like cells [52]. Surface wetting behavior is also known to influence the polarization and adhesion of macrophages to material surfaces. Indeed, the increased hydrophobicity observed after Cu-electroplating may have contributed to the increased CD86 production (M1 marker) compared to the Control-Ti. Additionally, this hydrophobicity may have further contributed to the reduction in macrophages adhered to the titanium. Indeed, we found that differentiation towards the two lineages was comparably high when THP-1 cells were treated with Cu-Ep-Ti conditioned media, but when cultured directly on the material surface, differentiation towards M2 – identified by the increased expression and presence of CD206 and MRC1, respectively – was notably greater than that of the M1. Together, it is possible that the presence of copper in a material such as Ti6Al4V-ELI could increase both the phagocytic rate and function of implant-local macrophages, while also modulating the inflammatory/healing environment coordinated by M1/M2 populations. This is an important factor in biomaterial design as orthopedic device implantation is already an inflammatory trigger, thus, it is vital to tune the immune cell response to avoid inducing excessive inflammation, promoting chronic inflammation, encapsulation of the implanted device, and increasing the risk of implant failure.

The data presented in this study indicates that the currently applied concentration of Cu was relatively high when the cytocompatibility was tested in in vitro static conditions. However, the antibiofilm and antimicrobial outcomes obtained in conjunction with the increase in macrophage-mediated phagocytosis represent a positive proof of concept worth of pursuing further. The proposed future steps would be fine-tuning the Cu content focusing on promoting the host-driven infection clearance using more complex models, such as co-cultures and in vivo infection models, as well as in combination with clinically relevant antibiotics.

## Conclusion

In conclusion, electrochemical deposition of metallic copper onto Ti6Al4V-ELI reduced the viability of *S. aureus* and prevented its colonization of the material surface, whilst simultaneously promoting clearance by macrophages through increased phagocytosis. This dual function, acting as both antimicrobial and immunostimulatory material, suggests that Cu-Ep-Ti could be of translational interest for future orthopedic devices. Further investigations should build upon these data and investigate the ability of Cu-Ep-Ti to prevent colonization and infection with other clinically relevant pathogens, as well as long-term in vivo studies to pursue a future translational application.

## Supplementary information

Supplementary Information
