# Supplementary material for: Anodized Ti6Al4V-ELI, electroplated with copper is bactericidal against Staphylococcus aureus and enhances macrophage phagocytosis
Source: J Mater Sci Mater Med. 2025 Jan 24;36(1):14. doi: 10.1007/s10856-024-06853-4 (PMC11761993; doi:10.1007/s10856-024-06853-4)
Supplement: Supplementary file 1 — Supplementary Information [file 10856_2024_6853_MOESM1_ESM.docx]

**Anodized Ti6Al4V-ELI, electroplated with copper is bactericidal against *Staphylococcus aureus* and enhances macrophage phagocytosis**

Paula Milena Giraldo-Osorno^1, 2, †^, Adam Benedict Turner^1, 2, †^, Sebastião Mollet Barros ^3, 4 †^, Robin Büscher ^3^, Simone Guttau ^3^, Farah Asa’ad ^1,5^, Margarita Trobos^1, 2,^ *, Anders Palmquist^1,^ *

^†^ Equal contribution; MT and AP are joint last authors

^1^ Department of Biomaterials, Institute of Clinical Sciences, Sahlgrenska Academy, University of Gothenburg, Gothenburg, Sweden

^2^ Centre for Antibiotic Resistance Research in Gothenburg (CARe), University of Gothenburg, Gothenburg, Sweden

^3^ Stryker Trauma Gmbh, Schönkirchen, Germany,

^4^ Faculty of Medicine, Centre for Translational Bone, Joint and Soft Tissue Research, Technische Universität Dresden, Germany.

^5^ Department of Oral Biochemistry, Institute of Odontology, Sahlgrenska Academy, University of Gothenburg, Gothenburg, Sweden

***Correspondence:**

Anders Palmquist: [anders.palmquist@biomaterials.gu.se](mailto:anders.palmquist@biomaterials.gu.se)

Margarita Trobos: [margarita.trobos@biomaterials.gu.se](mailto:margarita.trobos@biomaterials.gu.se)

Box 412

SE-40530 Gothenburg

Sweden

**Supplementary methods**

hFOB cell culture conditions

The human osteoblast cell line, hFOB 1.19 (ATCC CRL-11372, Manassas, VA, USA), was cultured in α-MEM with glutamax (Gibco Life Technologies, Waltham, MA, USA) medium. This medium was supplemented with 10 % HI-FBS, 1 % penicillin/streptomycin (PEST) solution (both from Gibco Life Technologies). The cell culture was maintained in a 37 °C humidified incubator with 5 % CO_2_. Cells from the third passage were used.

Resin embedding of macrophages

The procedure involves controlled thin-resin plasticization of adherent cells and bacteria with heavy metal staining, preserving subcellular structures while providing support for FIB milling. The method consisted of five main steps: cell fixation, heavy metal staining, resin infiltration, extracellular resin removal, and resin polymerization.

Sample preparation included co-culturing THP-1 cells and *S. aureus* bacteria on the discs. Post 24-h incubation, the cells are fixed using formaldehyde and stained using the RO-T-O (reduced osmium-thiocarbohydrazide-osmium) procedure and en bloc staining with uranyl acetate. After fixation and staining, cells are dehydrated in a graded ethanol series and infiltrated with an epoxy-based resin to preserve their structure. The samples were gold sputtered prior analysis in a dual beam FIB/SEM (FEI Versa3D).

**Supplementary figures**

**Figure S1.** Acidification control for phagocytosis of *S. aureus* bioparticles.

***Positive control:*** *THP-1 macrophages;* ***Cu-Ep Ti CM:*** *24 h conditioned media from pre-leached Cu-Ep Ti (No cells);* ***Control Ti CM:*** *24 h conditioned media from pre-leached Control Ti (No cells);* ***Cu-Ep Ti CM Cell acidification control:*** *THP-1 macrophages grown 24 h in conditioned media from pre-leached Cu-Ep Ti. One centrifugation step was added to remove cells.*


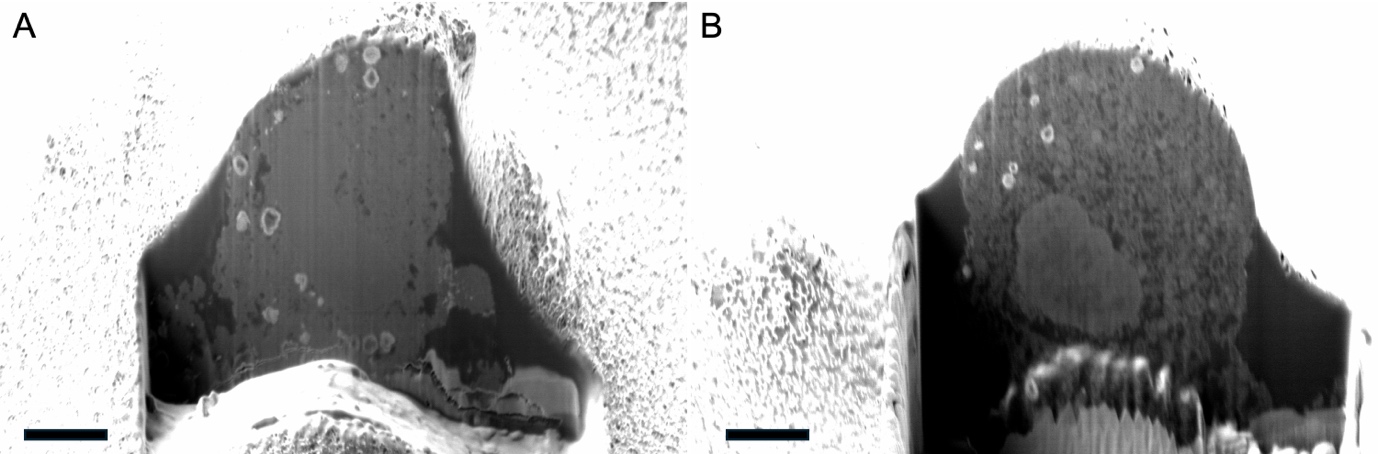


**Figure S2.** Cross-sectional view of a macrophages on A) Control-Ti and B) Cu-Ep Ti showing phagocytosis of pHrodo bioparticles. Scale bars = 5 µm

**Figure S3.** Expression of the CD14 surface marker before and after differentiation with PMA.

Blue: DAPI; Green: CD14 surface marker


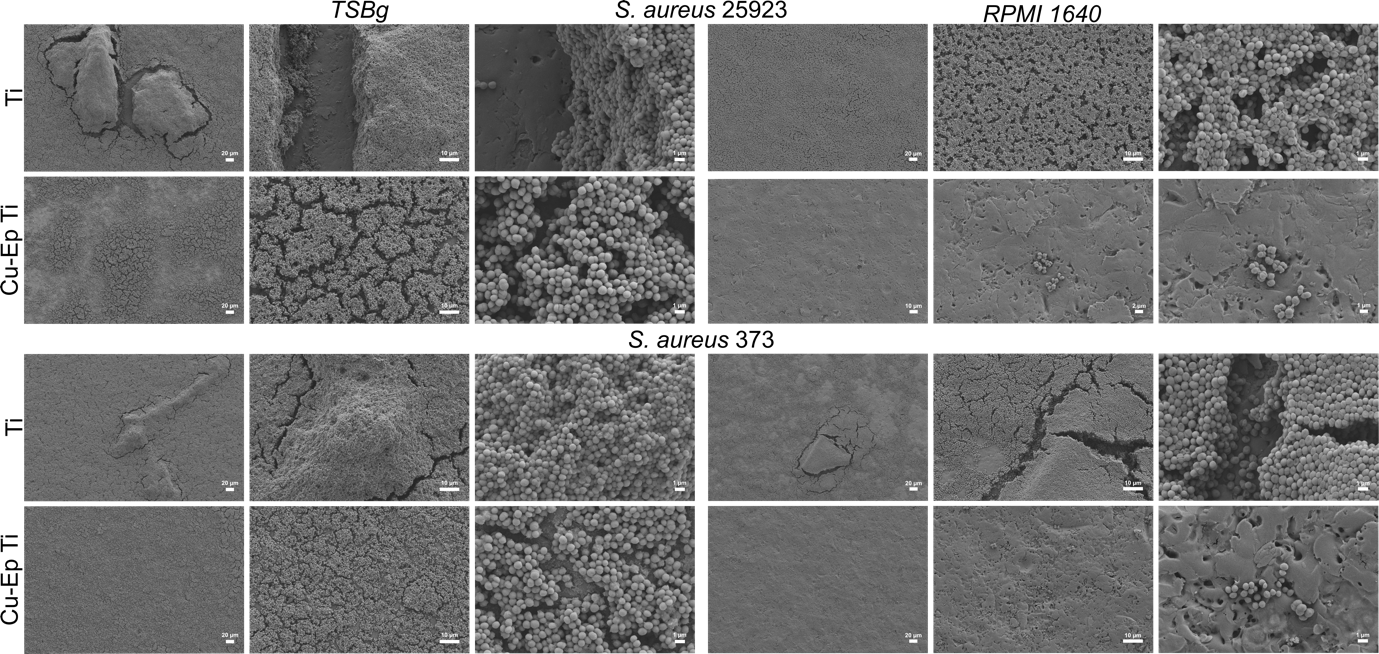


**Figure S4**. Comparative SEM images of the biofilms formed on the Ti and Cu-Ep-Ti in TSBg and RPMI 1640 with both bacterial strains.


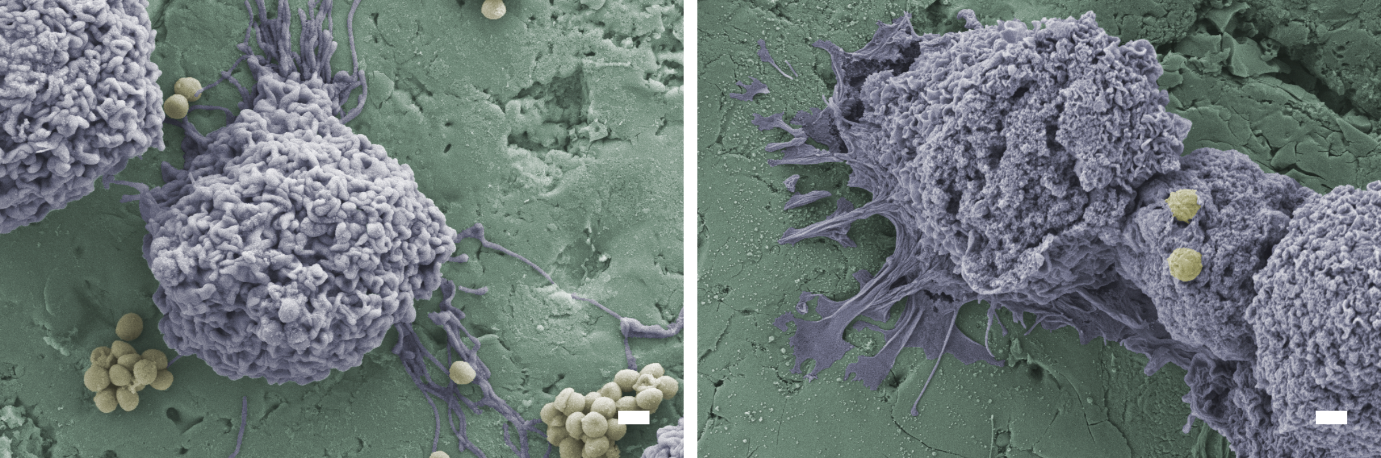


**Figure S5**. Pseudo-colored SEM micrographs of THP-1 macrophages (blue) grown on either **A)** Control Ti or **B)** Cu-Ep-Ti samples with pHrodo *S. aureus* bioparticles.

Blue = THP-1 macrophage; Yellow = pHrodo *S. aureus* bioparticle; Green = Control-Ti (A) and Cu-Ep-Ti (B)

Scale bar = 1 µm


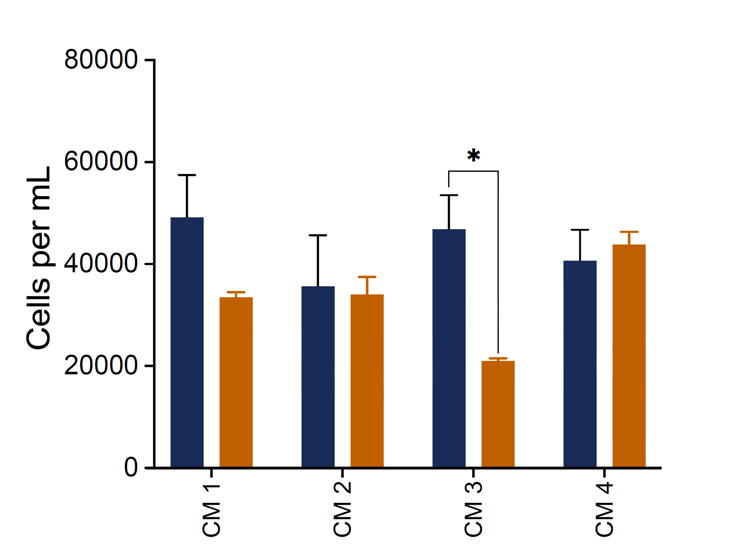


**Figure S6.** Number of viable osteoblasts after 24 h interaction with copper conditioned media. Copper concentration in conditioned media from Cu-Ep-Ti, CM1: 702 ± 96 μM; CM2: 402 ± 71 μM; CM3: 857 ± 370 μM; and CM4: 369 ± 168 μM.
